# Increased secretion of adipocyte-derived extracellular vesicles is associated with adipose tissue inflammation and the mobilization of excess lipid in human obesity

**DOI:** 10.1186/s12967-024-05249-w

**Published:** 2024-07-04

**Authors:** Johanna Matilainen, Viivi Berg, Maija Vaittinen, Ulla Impola, Anne-Mari Mustonen, Ville Männistö, Marjo Malinen, Veera Luukkonen, Natalia Rosso, Tanja Turunen, Pirjo Käkelä, Silvia Palmisano, Uma Thanigai Arasu, Sanna P. Sihvo, Niina Aaltonen, Kai Härkönen, Andrea Caddeo, Dorota Kaminska, Päivi Pajukanta, Minna U. Kaikkonen, Claudio Tiribelli, Reijo Käkelä, Saara Laitinen, Jussi Pihlajamäki, Petteri Nieminen, Kirsi Rilla

**Affiliations:** 1 Faculty of Health Sciences, School of Medicine, Institute of Biomedicine, University of Eastern Finland, P.O. Box 1627, 70211 Kuopio, Finland; 2 Institute of Public Health and Clinical Nutrition, University of Eastern Finland, Kuopio, Finland; 3 Finnish Red Cross Blood Service, Helsinki, Finland; 4 Faculty of Science, Forestry and Technology, Department of Environmental and Biological Sciences, University of Eastern Finland, Joensuu, Finland; 5 Kuopio University Hospital, Kuopio, Finland; 6 Faculty of Health Sciences, School of Medicine, Institute of Clinical Medicine, University of Eastern Finland, Kuopio, Finland; 7 Department of Forestry and Environmental Engineering, South-Eastern Finland University of Applied Sciences, Kouvola, Finland; 8 Metabolic Liver Disease Unit, Centro Studi Fegato, Fondazione Italiana Fegato, SS14 Km 163.5 Area Science Park Basovizza, Trieste, Italy; 9 Faculty of Health Sciences, School of Medicine, Institute of Clinical Medicine, Department of Surgery, University of Eastern Finland, Kuopio, Finland; 10 Surgical Clinic Division, Cattinara Hospital, Trieste, Italy; 11 Department of Medical, Surgical and Health Sciences, University of Trieste, Trieste, Italy; 12 Faculty of Health Sciences, A. I. Virtanen Institute for Molecular Sciences, University of Eastern Finland, Kuopio, Finland; 13 Faculty of Biological and Environmental Sciences, Molecular and Integrative Biosciences Research Programme, University of Helsinki, Helsinki, Finland; 14 Helsinki Institute of Life Science (HiLIFE), Helsinki University Lipidomics Unit (HiLIPID), Biocenter Finland, Helsinki, Finland; 15 Department of Molecular and Clinical Medicine, Institute of Medicine, Wallenberg Laboratory, University of Gothenburg, Sahlgrenska Academy, Gothenburg, Sweden; 16 Department of Medicine, Division of Cardiology, David Geffen School of Medicine at UCLA, Los Angeles, CA USA; 17 Department of Human Genetics, David Geffen School of Medicine at UCLA, Los Angeles, CA USA; 18 Institute for Precision Health, David Geffen School of Medicine at UCLA, Los Angeles, CA USA; 19 Endocrinology and Clinical Nutrition, Kuopio University Hospital, Kuopio, Finland; 20 Faculty of Science, Forestry and Technology, Department of Technical Physics, University of Eastern Finland, P.O. Box 1627, 70211 Kuopio, Finland

**Keywords:** Adipocyte, Adipose tissue, Extracellular vesicles, Fatty acids, Inflammation, Obesity

## Abstract

**Background:**

Obesity is a worldwide epidemic characterized by adipose tissue (AT) inflammation. AT is also a source of extracellular vesicles (EVs) that have recently been implicated in disorders related to metabolic syndrome. However, our understanding of mechanistic aspect of obesity’s impact on EV secretion from human AT remains limited.

**Methods:**

We investigated EVs from human Simpson Golabi Behmel Syndrome (SGBS) adipocytes, and from AT as well as plasma of subjects undergoing bariatric surgery. SGBS cells were treated with TNFα, palmitic acid, and eicosapentaenoic acid. Various analyses, including nanoparticle tracking analysis, electron microscopy, high-resolution confocal microscopy, and gas chromatography–mass spectrometry, were utilized to study EVs. Plasma EVs were analyzed with imaging flow cytometry.

**Results:**

EVs from mature SGBS cells differed significantly in size and quantity compared to preadipocytes, disagreeing with previous findings in mouse adipocytes and indicating that adipogenesis promotes EV secretion in human adipocytes. Inflammatory stimuli also induced EV secretion, and altered EV fatty acid (FA) profiles more than those of cells, suggesting the role of EVs as rapid responders to metabolic shifts. Visceral AT (VAT) exhibited higher EV secretion compared to subcutaneous AT (SAT), with VAT EV counts positively correlating with plasma triacylglycerol (TAG) levels. Notably, the plasma EVs of subjects with obesity contained a higher number of adiponectin-positive EVs than those of lean subjects, further demonstrating higher AT EV secretion in obesity. Moreover, plasma EV counts of people with obesity positively correlated with body mass index and *TNF* expression in SAT, connecting increased EV secretion with AT expansion and inflammation. Finally, EVs from SGBS adipocytes and AT contained TAGs, and EV secretion increased despite signs of less active lipolytic pathways, indicating that AT EVs could be involved in the mobilization of excess lipids into circulation.

**Conclusions:**

We are the first to provide detailed FA profiles of human AT EVs. We report that AT EV secretion increases in human obesity, implicating their role in TAG transport and association with adverse metabolic parameters, thereby emphasizing their role in metabolic disorders. These findings promote our understanding of the roles that EVs play in human AT biology and metabolic disorders.

**Supplementary Information:**

The online version contains supplementary material available at 10.1186/s12967-024-05249-w.

## Background

Globally, obesity affects 13% of the total world adult population [1], with developed countries like the USA showing a 36% adult obesity rate [2]. This condition triggers chronic low-grade inflammation, macrophage accumulation, and dysregulated production of inflammatory cytokines in adipose tissue (AT) [3], leading to local and systemic insulin resistance (IR) and associated pathologies. AT, beyond storing energy as triacylglycerol (TAG)-filled lipid droplets, secretes various cytokines, hormones, and adipokines regulating whole-body energy metabolism. Recently, AT has been demonstrated to secrete extracellular vesicles (EVs) in high quantities [4]. These EVs, originating from plasma membrane or endosomal compartments of cells, serve as essential mediators between cells and tissues, carrying and transferring all types of biomolecules from their parental cells, including lipids, proteins, nucleic acids, and sugars [5].

Previous studies have shown elevated levels of circulating EVs in obesity and obesity-related conditions, including IR, diabetes, and non-alcoholic fatty liver disease [6–9]. There is evidence that the majority of circulating EVs may originate from AT [10]. More detailed in vitro investigations have often utilized murine models, particularly mouse 3T3-L1 adipocytes. Several obesity-associated events, including tumor necrosis factor α (TNFα)-induced inflammation, hypoxia, and palmitic acid (PA, 16:0) exposure, promote EV secretion from these cells [11–13]. Indeed, the number of secreted EVs from subcutaneous AT (SAT) and visceral AT (VAT) increased in obese compared to lean mice [4]. Despite these findings, detailed, mechanistic studies on the effects of obesity on EV secretion from the AT of human origin remain scarce. Patient omental and SAT EV counts from ex vivo cultures have been shown to correlate positively with homeostatic model assessment for IR (HOMA-IR) [14] and body mass index (BMI) [4], respectively. An elegant study by Camino et al. [15] suggested that in obesity and type 2 diabetes (T2D) the amounts of AT EVs increase in human circulation. The idea was based on the high quantity of transforming growth factor β 1 (TGFB1) and mimecan in AT EVs of subjects with obesity, as well as elevated levels of TGFB1- and mimecan-containing EVs in the plasma of subjects with obesity and diabetes. Intriguingly, AT EVs might contribute to TAG release, as supported by the presence of TAGs in mouse AT EVs, and their doubled amount in AT EVs from obese mice compared to those from lean mice [16, 17]. These suggest that EV secretion may be another important mechanism for AT to mobilize TAGs into circulation.

Previous studies have largely concentrated on the proteomic and miRNA analysis of rodent adipocyte or AT EVs [10, 11, 18]. A few recent reports examined SAT and VAT EV proteomic profiles, revealing greater IR- and inflammation-related proteins in VAT EVs [15, 19]. Regarding lipid profiles, murine 3T3-L1 cell-derived small and large EVs’ lipid classes have been quantified [11]. Other studies have found distinct fatty acid (FA) profiles in pre- and mature 3T3-L1 cell EVs [20], and the presence of arachidonic acid (ARA, 20:4n-6), eicosapentaenoic acid (EPA, 20:5n-3), docosahexaenoic acid (DHA, 22:6n-3), and several inflammation-related lipid mediators in mature 3T3-L1 EVs under PA exposure [21]. Moreover, a recent report elucidates the FA profiles of VAT EVs from two mouse obesity models, demonstrating differing lipid class proportions and higher amount of the lipid species containing ARA and stearic acid (SA, 18:0) [22]. However, to the best of our knowledge, detailed FA profiles of EVs from human AT or adipocytes have not been documented previously.

In the present study, we aimed to investigate EV secretion from adipocytes and AT in human obesity by utilizing Simpson Golabi Behmel Syndrome (SGBS) adipocyte cell strain and patient AT ex vivo cultures, respectively. Particularly, great effort was put into detailed EV validations to provide reliable data for the research field, which is in its early stages. FA profiles of EVs from patient VAT and SAT, as well as from SGBS cells after exposure to TNFα, PA, and EPA, were investigated thoroughly. AT EV secretion into circulation was studied by analyzing plasma EVs from normal-weight controls and subjects with obesity by imaging flow cytometry. Furthermore, we utilized high-resolution confocal microscopy to investigate the possible presence of TAGs in EVs from AT and adipocytes. Based on previous reports on mice, we hypothesized that inflammatory components would trigger EV secretion from human adipocytes, and that enhanced AT EV secretion could also be realized in circulation. Additionally, we aimed to obtain further support for the role of AT EVs as TAG carriers.

## Methods

### Patient samples from bariatric surgeries for AT and blood collection

A total of 10 subjects with obesity (1 male and 9 females) undergoing Roux-en-Y gastric bypass operation were included for obtaining AT samples (Table 1). Of these, 8 individuals were participating in the ongoing Kuopio Obesity Surgery Study (KOBS) [23], and 2 in the study ongoing at the Italian Liver Foundation (Fondazione Italiana Fegato) in collaboration with the Surgery Department of the Secondary Care—Cattinara Hospital, Trieste, Italy. Anthropometric, clinical, and biochemical parameters were assessed at baseline, as previously described [24, 25]. Blood samples were drawn from another group of patients undergoing bariatric surgery at the Kuopio University Hospital, Kuopio, Finland, after a 12-h o/n fasting, before surgery. The study protocol in Kuopio was approved by the Ethics Committee of Northern Savo Hospital District (54/2005, 104/2008, 27/2010, and 1108/2018), and the one in Trieste by the Local Ethical Committee (protocol N. 22979, Comitato Etico Regionale Unico, Friuli Venezia Giulia, Sistema Sanitario Nazionale), and both were carried out in accordance with the Helsinki Declaration. Informed written consent was obtained from all participants.

**Table 1:** Clinical characteristics of the patients from whom fasting plasma and adipose tissue samples were obtained

	AT (n = 10)	Plasma (n = 10)
Gender (male/female)	1/9	5/5
Age (years)	47 ± 3.0	50 ± 3.0
Body mass index (kg/m^2^)	41.4 ± 1.70	39.8 ± 1.32
Triacylglycerols (mmol/l)	1.64 ± 0.42	1.49 ± 0.22
Total cholesterol (mmol/l)	5.4 ± 0.42	3.9 ± 0.31
HDL-cholesterol (mmol/l)	1.1 ± 0.08	1.0 ± 0.09
LDL-cholesterol (mmol/l)	3.6 ± 0.39	2.3 ± 0.28
Glucose (mmol/l)	6.4 ± 0.35	6.2 ± 0.34
Insulin (mU/l)	23.9 ± 9.03	11.5 ± 1.64

Mean ± SEM

*AT* adipose tissue, *HDL* high-density lipoprotein, *LDL* low-density lipoprotein

### AT collection, processing, and culturing

AT collection and ex vivo culture procedures were modified from [26]. After obtaining VAT (omental) and SAT (abdominal) biopsies by surgical resection, they were placed into a capped, sterile 50 ml Falcon tube containing either phosphate buffered saline (PBS) or AT ex vivo medium [(DMEM high glucose, either ECB7501L, EuroClone, Pero, Italy; or D6546, Sigma-Aldrich, St. Louis, MO, USA; containing 10% EV-depleted fetal bovine serum (FBS), 2 mM L-glutamine (ECB3000D, EuroClone), 100 IU/ml penicillin and 100 µg/ml streptomycin (ECB3000D, EuroClone), 10 µg/ml human insulin (I9278, Sigma-Aldrich), 1 µM dexamethasone (D4902, Sigma-Aldrich), and 50 µg/ml gentamicin (G1397, Sigma-Aldrich)]. EV depletion from FBS was performed by 110,000 × *g* centrifugation for 16 h at + 4 °C, and sterile-filtering with 0.22 µm syringe filters (Minisart, Sartorius Stedim Biotech, Göttingen, Germany). AT samples were transported to cell culture laminar hood as soon as possible for AT processing. AT was placed onto a sterile Petri dish and minced using sterile scalpel and forceps into around 1–2 mm^3^ pieces. Next, AT was transferred onto a pre-weighed Petri dish and weighed. AT minces were then placed onto a 500 µm nylon mesh (43-50300-01, pluriSelect Life Science, Leipzig, Germany) affixed on the top of a sterile 50 ml Falcon tube with forceps. Sterile PBS (RT) was added several times over the minced tissue to remove broken cell debris, lipids, and blood. Tissue pieces were carefully removed from the mesh with forceps, so that the waste would pass through the mesh. Visible blood clots were removed with forceps. AT was cultured on either 6-well plate, 10 or 15 cm dish according to weight: around 100 mg was cultured on each well of 6-well plate (AT ex vivo medium volume 3 ml), 300–500 mg on a 10 cm dish (medium volume 15 ml), and 700 mg on a 15 cm dish (medium volume 22.5 ml).

### Harvesting and purification of EVs from AT ex vivo medium

Conditioned media were collected from AT ex vivo cultures daily until 3 days from culture initiation had passed. Each time, fresh AT ex vivo medium was carefully added for cultures. After the medium collection on day 3, renewed AT ex vivo medium was incubated for 3 more days, after which the final (day 6) medium was collected. Media were carefully collected into 50 ml Falcon tubes on which a sterile 500 µm nylon mesh was placed. They were immediately centrifuged at 12,000 × *g* for 10 min at + 4 °C, to separate any large contaminants and to leave large oil droplets arising from broken adipocytes in a layer on top. The supernatant was carefully collected so that the upmost part, possibly containing lipid droplets from broken adipocytes, was discarded. Medium was filtered through a 5 µm syringe filter (Minisart 17594-K, Sartorius Stedim Biotech) to remove cell debris, and then stored at − 80 °C until EV isolation by serial ultracentrifugation. For EV isolation, medium was centrifuged at 183,000 × *g* at + 4 °C for 90 min. Supernatant was discarded and the pellet resuspended in sterile-filtered PBS (filtered with 0.22 µm syringe filter), after which an additional, similar ultracentrifugation as a washing step was performed. Finally, the remaining pellet was resuspended into sterile-filtered PBS and stored at − 80 °C until the further analysis.

### Human SGBS cell culture and differentiation

Human SGBS preadipocytes [27] were cultured in Dulbecco’s Modified Eagle Medium/Nutrient Mixture F-12 (DMEM/F-12) (11330057, Thermo Fisher Scientific, Vilnius, Lithuania) supplemented with 33 µM biotin (B4639, Sigma-Aldrich), 17 µM D-pantothenic acid hemicalcium salt (pantothenate) (P5155, Sigma-Aldrich), 100 U/ml penicillin and 100 µg/ml streptomycin (EuroClone), and 10% FBS (10270106, Life Technologies, Burlington, ON, Canada) until reaching confluence. Preadipocytes were induced to differentiate into mature adipocytes as previously described, with the exception that 3FC medium included 3% FBS [28].

### Conditioned medium collection from SGBS adipocytes

For medium collection and EV isolation from preadipocytes, cells that had reached confluence were washed with PBS, after which growth medium with 10% EV-depleted FBS was added for 24 h. For medium collection from mature adipocytes, mature SGBS cells were washed with PBS, after which 3% EV-depleted FBS 3FC medium was added for 24 h.

Prior to experiments and medium collection for EV isolation, mature SGBS cells were washed with PBS, after which 3% EV-depleted FBS 3FC medium supplemented with desired components was added. Cells were treated with either TNFα (300-01, PeproTech, Rocky Hill, NJ, USA), PA (P9767, Sigma-Aldrich), or EPA (90110, Cayman Chemical, Ann Arbor, MI, USA) for 24 h. When preparing treatment media for PA and EPA treatments, FAs were complexed with 10% FA-free bovine serum albumin (BSA) in PBS with the FA:BSA molar ratios of 4:1 and 2.7:1, respectively [29, 30]. The BSA concentration residing in FBS was taken into consideration. Cells treated with the appropriate vehicle, without TNF*α*, PA, or EPA, were used as controls.

### Isolation of EVs from SGBS adipocyte conditioned media

For EV isolation, conditioned media from SGBS adipocytes were first filtered through a 5 µm syringe filter to remove cell debris, and then stored at − 80 °C until EV isolation by standard ultracentrifugation. Media were first centrifuged at 10,000 × *g* for 90 min at + 4 °C, and the remaining pellets of large EVs were resuspended in sterile-filtered (0.22 µm syringe filters) PBS. The supernatant was further centrifuged at 183,000 × *g* at + 4 °C for 90 min in order to pellet the smallest EVs, and these fractions were then pooled with the fractions of large EVs. The final EV-preparations were stored at − 80 °C until the further analysis.

### Real-time quantitative PCR (RT-qPCR)

After treatments, SGBS cells were lysed with TRI Reagent® (Molecular Research Center, Cincinnati, OH, USA) for mRNA expression analyses. Total RNA extraction, cDNA synthesis, and RT-qPCR were performed as previously described [31]. Hypoxanthine phosphoribosyltransferase 1 was used as the reference gene. The primer sequences are shown in Table S1 (see Additional File 1). The data were expressed as fold changes compared to control.

### SDS–polyacrylamide gel electrophoresis (PAGE) and Western Blotting (WB)

Proteins were extracted using RIPA lysis buffer (150 mM sodium chloride, 50 mM Tris, 1% Nonidet, 0.1% SDS (Sigma-Aldrich), and 0.5% sodium deoxycholate in PBS, pH 7.5) supplemented with 1 mmol/l sodium orthovanadate, 0.1 mg/ml phenylmethylsulfonyl fluoride, and 300 U/ml aprotinin, and resolved by SDS-PAGE, followed by transfer to Amersham Protran nitrocellulose membrane (GE Healthcare, Chicago, IL, USA) with 350 mA current in Mini-PROTEAN® Tetra Blotting Module wet blotter (Bio-Rad Laboratories, Hercules, CA, USA) or with 2 mA/cm^2^ current in Fastblot B43 semidry blotter (Biometra, Göttingen, Germany). Membranes were incubated with primary antibodies (Table S2, see Additional File 1) at + 4 °C for o/n and imaged with ChemiDoc MP Imaging System (Bio-Rad Laboratories). Protein intensities and relative protein expression levels were quantified with the Image Lab software (Bio-Rad Laboratories).

### Glycerol assay

The glycerol concentrations were assessed from media obtained from TNFα-treated cells by free glycerol reagent (F6428, Sigma-Aldrich) and glycerol standard (G7793, Sigma-Aldrich), according to the manufacturer’s protocol but modified for 96-well plate format. Briefly, 5 µl of each standard and sample replicates were added into a 96-well plate, and 200 µl of Free glycerol reagent was further added.

### Blood sampling and isolation of EVs from plasma samples

Blood samples from normal-weight subjects (n = 4 males and 2 females; Table S3, see Additional File 1) and bariatric surgery patients (n = 10, Table 1), after o/n fasting, were collected into EDTA tubes on ice. Within 30 min after collection, blood samples were centrifuged at 1000 × *g* for 15 min at RT, and plasma was collected into 2 ml Eppendorf tubes and stored at − 80 °C. For EV isolation, samples were centrifuged at 2500 × *g* for 15 min at RT to pellet large impurities. Supernatants were transferred into new tubes and centrifuged at 5000 × *g* for 15 min at RT and subsequently placed into ultracentrifuge tubes on ice, and ice-cold, sterile-filtered PBS was added 1:1 (v/v). Samples were centrifuged at 100,000 × *g* for 2 h at + 4 °C (Optima L-90 K ultracentrifuge with 50.4 Ti fixed angle-rotor, Beckman Coulter, Brea, CA, USA). The supernatants were discarded, and the remaining pellets resuspended in sterile-filtered PBS. Similar ultracentrifugation was performed as a washing step. The supernatants were again carefully discarded, after which the pellets were resuspended in sterile-filtered PBS or RIPA lysis buffer. Samples were then stored at − 80 °C.

### Analysis of plasma EVs by flow cytometer

Plasma EV samples resuspended in sterile-filtered PBS were analyzed with 12 channel Amnis® ImageStream®X Mark II imaging flow cytometer (Luminex Corporation, Austin, TX, USA). Samples were labelled in 25 µl volume, with FITC-CD9 (312103, BioLegend, San Diego, CA, USA) and adiponectin antibodies (Anti-acrp30, SC-136131, Santa Cruz, Santa Cruz, CA, USA) in dark at RT for 60–90 min. The adiponectin antibody was labelled with Zenon AF647 fluorescent labelling kit (Z25008, Invitrogen, Life Technologies, Eugene, OR, USA) according to manufacturer’s protocols. The instrument and INSPIRE software were set up as follows: excitation lasers 488, 642, and 785 and channels 01 (Ch01, bright field, BF), Ch06 (scattering channel), plus fluorescence channels Ch02 and Ch011 were activated for signal detection. Amnis® High Gain Mode and 60 × magnification were used for enhanced small particle detection. Antibody staining was counted from both small EVs (particle diameters up to 150–200 nm) and from all particles (including particles with diameters up to 400–500 nm) separately. Sample buffer (PBS) and antibody only controls (anti-adiponectin and AF647, FITC-CD9) were used to determine background and auto fluorescent signals.

### Transcriptomic analyses of SAT specimens

RNA extraction and sequencing analysis of SAT were performed for the same patients from which plasma samples were obtained to allow the comparison of EV data to *TNF* expression. These samples belonged to the KOBS study that has been described in detail previously [32]. In brief, RNA sequencing libraries underwent 69-nucleotide long paired-end sequencing. These reads were subsequently mapped to the human reference genome (GRCh38/hg38) with Gencode 29 annotations using STAR aligner [33] in 2-pass mode. Following alignment, gene-level counts were normalized using the trimmed mean of M values method, then converted to counts per million with edgeR [34], and log-transformed for analysis. To enhance the quality of subsequent analyses, expression data were adjusted to control for technical factors and potential confounders, specifically, the percentage of uniquely aligned reads and 3’ bias.

### Lipoprotein isolation from serum samples

For obtaining reference material for assessing the purity of plasma EV samples, very-low-density lipoprotein (VLDL), low-density lipoprotein (LDL), and high-density lipoprotein (HDL) fractions were isolated at low salt concentrations in potassium bromide, as previously described [35]. Pure lipoprotein fractions were stored at – 80 °C.

### Nanoparticle tracking analysis (NTA)

The size distribution and concentration of particles in the EV preparations were analyzed using the Nanoparticle Tracking Analyzer (Malvern Panalytical, Malvern, UK) with a NS300 view unit, as previously described [36]. Data analysis was performed with the NTA 3.2 software (Malvern Panalytical).

### Electron microscopy (EM) analysis of EVs

The EV preparations resuspended in PBS were prepared for transmission electron microscopy (TEM) [37] and scanning electron microscopy (SEM) [36], as previously described.

### Imaging EVs by confocal microscopy

For imaging EVs by confocal microscopy, Ibidi chamber slides were coated with 10 μg/ml poly-D-lysine hydrobromide (P6407, Sigma-Aldrich) at + 37 °C in 5% CO_2_ for o/n. EVs were then placed on slides and incubated at + 37 °C in 5% CO_2_ for 3 h. Next, 1 h incubation with staining solution (1:200 AF594-CD63 (353033, BioLegend) and 1:200 FITC-CD9 (312103, BioLegend), or 1:200 AF594-CD63 and 1:1000 LipidSpot (LipidSpot™ 488 Lipid Droplet Stain, 70065 T, Biotium, Fremont, CA, USA) in 2% BSA PB) at RT was performed. The LipidSpot dye is a fluorescent neutral lipid stain used for staining intracellular lipid droplets, the main constituent of which is TAG. Adipocyte-derived EVs have been documented to contain also other neutral lipids, such as diacylglycerols, but with clearly lower levels [38]. Staining solution was removed, and sterile-filtered PBS was carefully added. EVs were visualized with 63 × NA 1.4 objective on a Zeiss Axio Observer inverted microscope equipped with a Zeiss LSM 800 confocal module.

### Gas chromatography–mass spectrometry

The FA profiles of EV samples, SGBS cells, and conditioned media were analyzed by gas chromatography–mass spectrometry. To obtain pre-SGBS cells, cells were washed with PBS, detached with trypsin, and centrifuged (1000 × *g* for 5 min). After centrifugation, cell pellets were stored at − 80 °C until the FA determinations. Mature SGBS cells were washed and then scraped. For the analyses, excess water was first removed from the subsamples of EVs and media by nitrogen stream followed by transmethylation in methanolic H_2_SO_4_ under nitrogen atmosphere [39]. The formed FA methyl esters (FAMEs) were extracted, the FAME and dimethyl acetal (DMA) structures identified, and the resulting chromatographic peaks integrated, as previously described [31]. The FA sums were calculated as the ∑(mol-%) of all individual FAs within a particular FA class, *i.e.*, saturated FAs (SFAs), monounsaturated FAs (MUFAs), polyunsaturated FAs (PUFAs), DMAs, and further for n-3 and n-6 PUFAs.

### Statistical analyses

Different statistical analyses were performed using the SPSS *v*27.0 software (IBM, Armonk, NY, USA). Comparisons between EV counts secreted by VAT and SAT were performed with the generalized linear model. Otherwise, comparisons with > 3 treatment or sample groups were conducted with the Kruskal–Wallis nonparametric analysis of variance (ANOVA). Nonparametric tests between two sample groups were performed with the Mann–Whitney U test. The *p-*value < 0.05 was considered statistically significant. The results are presented as the mean ± SEM. To perform a general assessment of the FA and DMA profiles of EVs, cells, or conditioned media, we also carried out supervised discriminant analyses (DA) by classifying the composition data by discriminant functions to see how samples in the different treatment groups differed from one another, which variables separated them most clearly, and how well the analysis was able to classify the samples into their respective groups.

## Results

### EVs from mature SGBS cells differ in size and quantity compared to ones from pre-SGBS cells

**Fig. 1:**
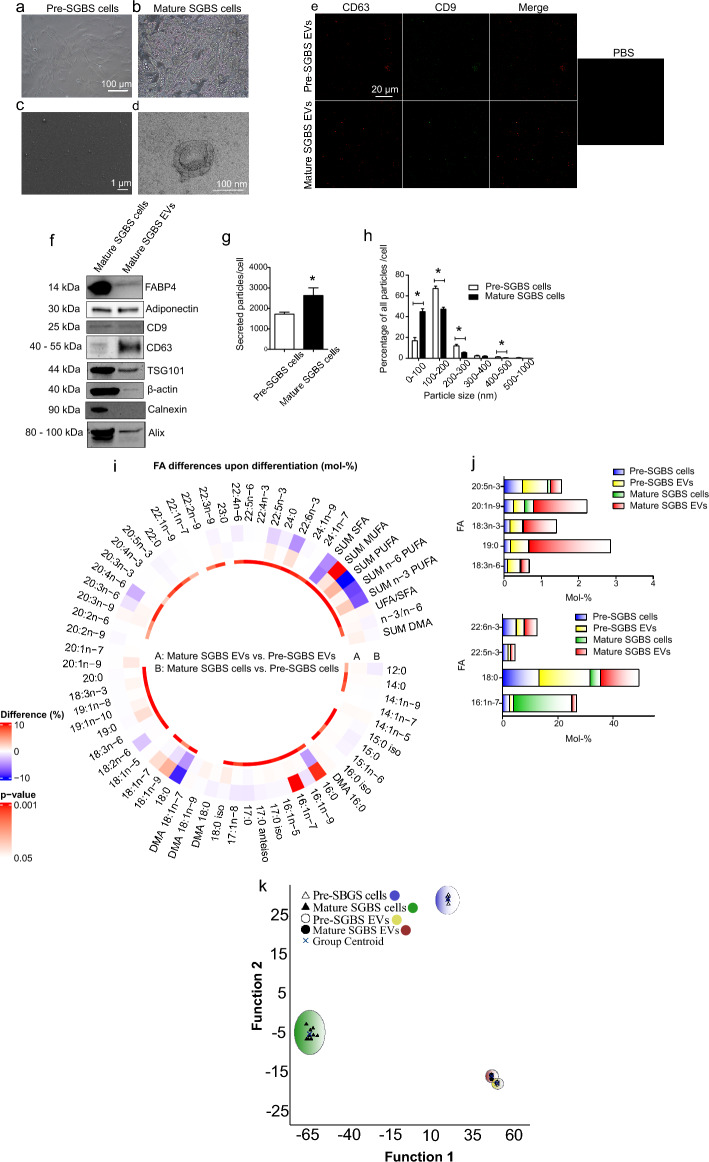
Characterization of extracellular vesicles (EVs) from Simpson Golabi Behmel Syndrome (SGBS) cells. The differentiation of pre-SGBS cells (**a**) into mature, lipid-laden SGBS cells was induced (**b**), after which EVs were isolated from culture medium by differential steps of ultracentrifugation. Scanning electron microscopy of EVs from mature SGBS cells (**c**) reveals good sample purity, and transmission electron micrograph a high-magnification image of a typical EV (**d**). The presence of EV-markers in EV samples was confirmed by fluorescent labelling of CD63 and CD9 in confocal microscopy (**e**). The presence of adipocyte-derived material (fatty acid binding protein 4 (FABP4) and adiponectin), tumor susceptibility 101 (TSG101), CD63, CD9, programmed cell death 6 interacting protein (Alix) and β-actin, as well as the absence of calnexin were further analyzed by Western Blotting from mature SGBS cell EV samples (**f**). Nanoparticle tracking analysis (NTA) of EV samples from pre- and mature SGBS cells revealed concentration (**g**) and size distribution (**h**) of secreted particles. NTA results are presented as mean + SEM, from 4 independent experiments. *p = 0.021 (Mann–Whitney U test). Differences in FA profiles of pre- and mature cells and their EVs were determined from total lipids with gas chromatography–mass spectrometry (**i**). Results are presented as percentage differences, calculated by subtracting the mol-% of each FA in the pre-group from the mol-% in the mature group. Red indicates an increase in the mature group, while blue indicates a decrease. *DMA* plasmalogen alkenyl chain-derived dimethyl acetal derivative, *SFA* saturated fatty acid, *MUFA* monounsaturated fatty acid, *PUFA* polyunsaturated fatty acid, unsaturated FA (UFA) = MUFA + PUFA. *p ≤ 0.05 Mann–Whitney U test *vs*. control. Percentages of selected FAs in pre- and mature cells and secreted EVs, presented as mean mol-% (**j**). The supervised discriminant analysis of FA proportions in pre- and mature SGBS cells, as well as their EVs (**k**)

The differentiation of preadipocytes (Fig. 1a) into lipid-laden adipocytes (Fig. 1b) was first induced, after which EVs were isolated from the conditioned medium. Confirming the presence of EVs and the sufficient sample purity, SEM and TEM analyses (Fig. 1c, d, respectively) revealed the presence of typical, cup-shaped EVs. The presence of EV-markers, CD63 and CD9 tetraspanins, was confirmed by fluorescent labeling and confocal microscopy (Fig. 1e). EV markers were further studied by WB, showing the presence of tumor susceptibility 101 (TSG101), CD63, CD9, programmed cell death 6 interacting protein (Alix), and β*-*actin, but also adipocyte-derived material, fatty acid binding protein 4 (FABP4) and adiponectin, in EVs from mature SGBS cells (Fig. 1f). Cross-contamination of EV samples with cell organelles was excluded by the absence of calnexin, an integral protein of the endoplasmic reticulum.

To investigate whether maturation into fully differentiated adipocytes changes EV secretion and particle size distribution, EVs obtained before and after differentiation were analyzed by NTA, demonstrating that mature SGBS cells secrete more EVs than pre-SGBS cells (Fig. 1g). These results indicate that adipogenesis, which is involved in the expansion of AT, would increase EV secretion from human AT. Moreover, size distribution analysis revealed that differentiation of the cells led to the secretion of smaller particles (averages for all particles: 150 ± 9.3 nm and 132 ± 8.6 nm from pre-SGBS and mature SGBS cells, respectively) (Fig. 1h). Gas chromatography–mass spectrometry analyses detected several FAs both in cells and EVs (Supplementary Figure S1, see Additional File 1). FAs with the highest proportions in pre- and mature SGBS cells as well as in their EVs were PA, palmitoleic acid (PLA, 16:1n-7), SA, oleic acid (OA, 18:1n-9), and *cis*-vaccenic acid (CVA, 18:1n-7), indicating that the FA profiles of EVs reflect well the profiles of the corresponding cells. As expected, the differentiation process caused changes in the FA proportions of cells (Fig. 1i). For instance, PA and PLA mol-% were remarkably higher in the mature SGBS adipocytes compared to the SGBS pre-adipocytes, while the proportions of several FAs, including SA, linoleic acid (LA, 18:2n-6), EPA, DHA, and total PUFAs decreased along with the differentiation.

There were also indications of different FA proportions between the EV types, although most of these changes did not reach statistical significance: PLA proportion was significantly higher in EVs from the mature cells compared to pre-SGBS EVs, and α-linolenic acid (ALA, 18:3n-3), 19:0, and 20:1n-9 tended to have higher average values in EVs from the mature cells compared to EVs from the pre-SGBS cells, while *γ*-linolenic acid (GLA, 18:3n-6) proportions seemed to be higher in pre-SGBS EVs (Fig. 1j). Nonetheless, the proportion of particular FAs in relation to their parental cells was different and, for example, SA, ALA, EPA, docosapentaenoic acid (DPA, 22:5n-3), and DHA were more abundant in EVs from mature SGBS cells than in mature SGBS cells. Indeed, despite the reduced proportion of PUFAs in SGBS cells along with the differentiation, this trend was reversed in the EVs, where PUFAs, particularly n-3 PUFAs, exhibited an inclination to increase (Fig. 1i).

The supervised DA of FA profiles showed that pre-SGBS and mature SGBS cells clustered apart from each other, and also from their EVs (Fig. 1k). However, EVs from pre- and mature SGBS cells were close to each other. Function 1, depicted on the horizontal axis, particularly separated mature SGBS cells, and function 2 pre-SGBS cells from other groups. Together these two functions explained 99.4% of the variance, and FAs mainly responsible for the separation were ARA, 24:0, LA, 24:1n-9, 14:0, 20:3n-3, ALA, SA, PA, EPA, dihomo-γ-linolenic acid (DGLA, 20:3n-6), DPA, 20:4n-3, 20:1n-7, DHA, 22:0, 12:0, OA, and 20:2n-6.

### Treatment of SGBS cells with TNFα, PA, and EPA reveal inflammation-triggered EV secretion and differing FA profiles of EVs

**Fig. 2:**
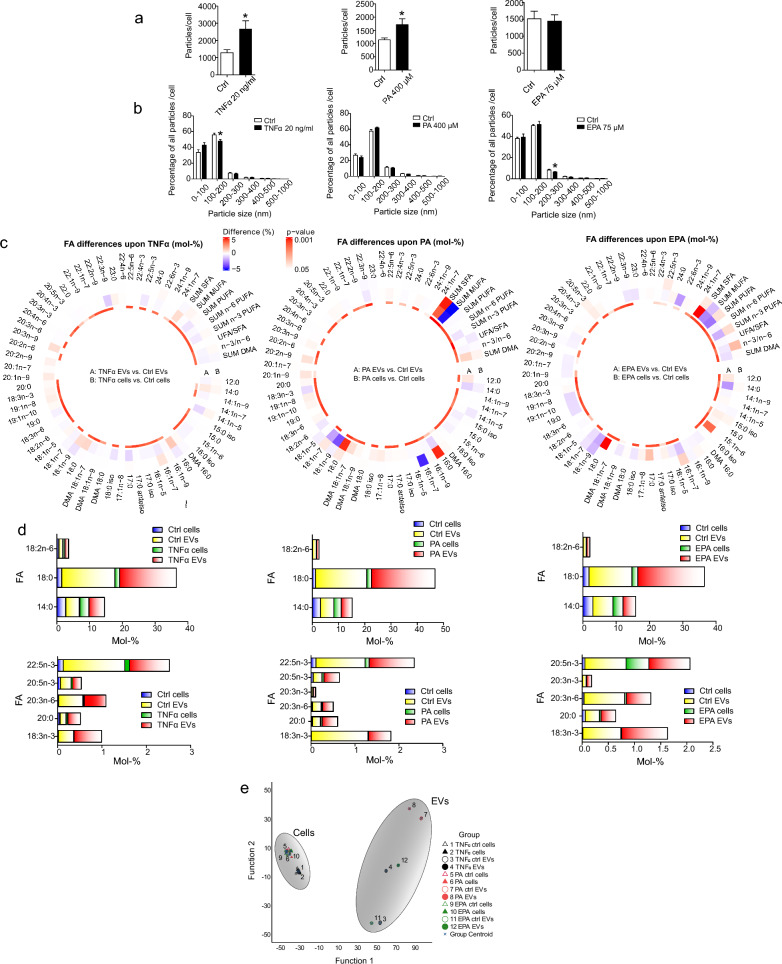
Studying extracellular vesicle (EV) secretion and EV fatty acid (FA) profiles from adipocyte treatments. Mature Simpson Golabi Behmel Syndrome cells were treated with either 20 ng/ml of tumor necrosis factor α (TNFα), 400 µM of palmitic acid (PA, 16:0) or 75 µM eicosapentaenoic acid (EPA, 20:5n-3) for 24 h, after which EVs were isolated and analyzed by nanoparticle tracking analysis (NTA). Both particle counts (**a**) and size distribution of particles (**b**) were obtained by nanoparticle tracking analysis NTA. Particle counts have been normalized to cell number, and results are presented as mean + SEM. *p < 0.05 (Mann–Whitney U test). Differences in FA profiles of cells and secreted EVs from TNFα, PA, and EPA treatments were determined from total lipids with gas chromatography–mass spectrometry (**c**). Results are presented as percentage differences, calculated by subtracting the mol-% of each FA in the control group from the mol-% in the treatment group. Red indicates an increase in the treatment group, while blue indicates a decrease. FAs are listed in the order of increasing chromatographic retention time. *DMA* plasmalogen alkenyl chain-derived dimethyl acetal derivative, *SFA* saturated fatty acid, *MUFA* monounsaturated fatty acid, *PUFA* polyunsaturated fatty acid, unsaturated FA (UFA) = MUFA + PUFA. *p ≤ 0.05 Mann–Whitney U test *vs*. control. Percentages of selected FAs in cells and secreted EVs from TNFα, PA, and EPA treatments, presented as mean mol-% (**d**). The FA results of TNFα and PA treatments were measured from 5 independent experiments and the results of EPA treatments from 7 independent experiments. The supervised discriminant analysis depicts the classification of FA signatures of cells and EVs from TNFα, PA, and EPA experiments based on discriminant functions 1 and 2 (**e**). Function 1 (on the *x*-axis) explained 81.7% of the variance in the dataset, and Function 2 10.6% of the variance

To investigate how factors associated with AT inflammation in human obesity affect EV secretion from adipocytes, we treated mature SGBS cells with TNFα, an inflammatory cytokine elevated in AT in obesity due to macrophage infiltration, pro-inflammatory PA, and anti-inflammatory EPA, to assess the effects of different exposures. Our preliminary experiments with increasing concentrations of TNFα and PA confirmed that they triggered inflammatory responses at the mRNA level (Supplementary Figure S2, See Additional File 1). For further experiments, we chose the concentrations which induced the expression of inflammation-related genes without a significant degree of cell death (20 ng/ml and 400 µM for TNFα and PA, respectively) (Supplementary Figure. S3, See Additional File 1). The anti-inflammatory effects of EPA were also confirmed by RT-qPCR (Supplementary Figure S4, See Additional File 1). Because the expression of several inflammation-related genes decreased after 75 µM treatment, this concentration was chosen for the actual experiments. NTA analyses of total particle counts indicated that inflammatory responses caused by TNFα and PA triggered EV secretion from adipocytes, while EPA treatment did not cause any change (Fig. 2a). Size distribution analyses revealed only a few significant changes in size distributions of secreted EVs after treatments (Fig. 2b).

Gas chromatography–mass spectrometry analyses of TNFα-*,* PA-, and EPA-treated cells and their EVs revealed that EVs and cells were well clustered in terms of FA profiles (Supplementary Figure S5, See additional file 1). Regarding FA modifications in cells, PA and EPA induced expected changes—PA increased PA and total SFA proportions and decreased unsaturated FA/SFA ratios, while EPA treatment resulted in higher percentages of DPA, total n-3 PUFAs, and elevated n-3/n-6 PUFA ratios (Fig. 2c). TNFα, on the other hand, did not significantly change cellular FA profiles. Remarkably, when comparing EVs and cells across all treatments, the observed differences in FA proportions were more prominent in EVs than in cells.

Differences between EV and cell FA profiles were also observed in DA, revealing that EVs and cell sample groups were clustered visibly apart from each other, indicating specific sorting of FAs into EVs (Fig. 2e). Interestingly, different EV types displayed three subgroupings, each consisting of two EV types. These were PA control and PA treatment EVs, TNFα and EPA EVs, as well as TNFα control and EPA control EVs. Different cell groups, on the other hand, were not separated that well from each other. The individual FAs most strongly separating the sample groups were, *e.g.*, LA, 14:1n-9, GLA, DGLA, 24:0, 24:1n-9, and 20:4n-3. From these, several FAs were more abundant in EVs when compared to parental cells, for instance, 14:0, SA, LA, DGLA, ALA, 20:0, 20:3n-3, EPA, and DPA (Fig. 2d). On the contrary, CVA, 14:1n-5, and 16:1n-9 proportions were or tended to be smaller in EVs *vs*. cells.

### Detailed characterization of patient AT EVs from ex vivo cultures

**Fig. 3:**
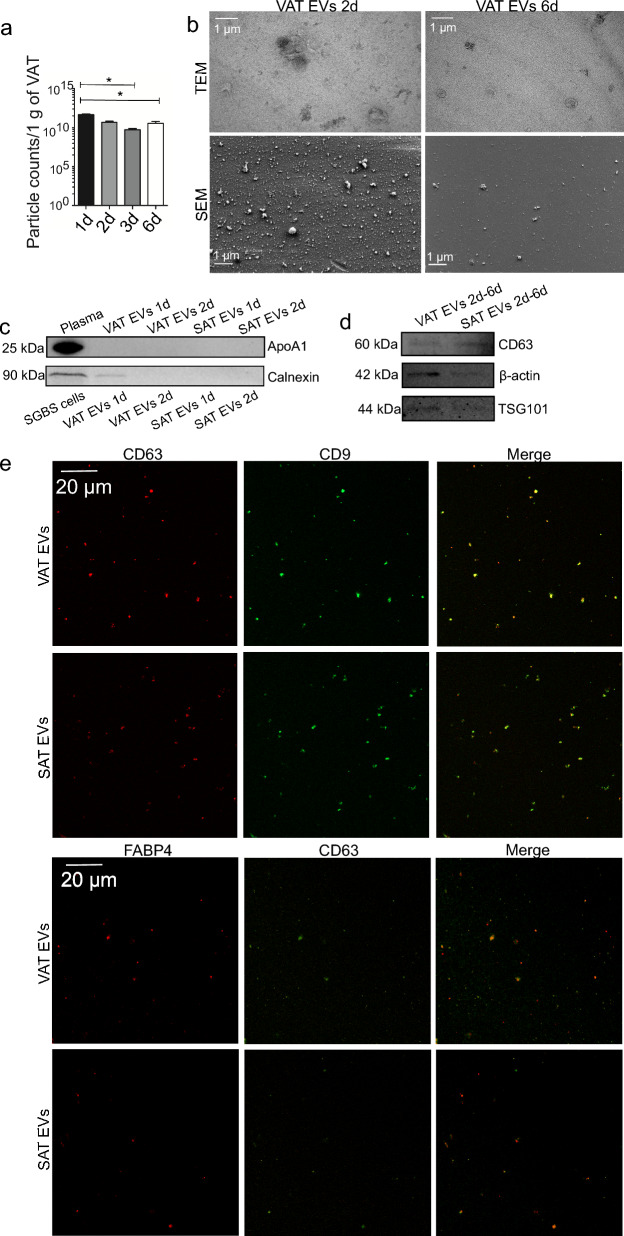
Characterization of patient visceral (VAT) and subcutaneous adipose tissue (SAT) extracellular vesicles (EVs). AT samples from bariatric surgeries were cultured ex vivo in EV-free culture medium for several days, after which EVs were isolated by differential steps of ultracentrifugation. To evaluate how EV secretion changes over time in AT cultures, culture supernatant was collected from VAT cultures and replenished daily until cultures had been maintained for 3 days in total (samples 1d, 2d, 3d). Replenished culture medium was then incubated for 3 more days, and then collected (6d). Results include VAT ex vivo cultures of 6 patients, presented as mean + SEM (**a**). *p = 0.006 (Kruskal–Wallis ANOVA). All EV counts have been normalized to 1 g of VAT obtained for culturing. Sample purity and the morphology of VAT EV isolates obtained after 2 and 6 days of culture initiation were analyzed by scanning (SEM) and transmission electron microscopy (TEM) (**b**). Scale bars 1 µm. The possible presence of blood- and cell-derived material was studied by apolipoprotein A1 (ApoA1) and calnexin Western Blotting, respectively, from 1 and 2d AT EV samples (**c**). Final VAT and SAT EV samples (pooled 2d, 3d, and 6d samples) were further analyzed by CD63, β-actin, and tumor susceptibility 101 (TSG101) Western Blotting (**d**). The presence of common EV markers (CD63 and CD9) and AT-specific EV marker fatty acid binding protein 4 (FABP4) was further confirmed by fluorescent labelling and confocal microscopy (**e**)

Regarding patient VAT and SAT samples, we first confirmed that no major degree of apoptosis was present in AT cultures by preparing histological sections for immunohistochemical and immunofluorescent staining for caspase-9, an upstream caspase involved in apoptosis (Supplementary Figure S6, see Additional File 1). NTA of EV samples at different culture timepoints revealed that particle counts decreased over time, suggesting a diminished secretory function of AT when cultured ex vivo for a prolonged time (Fig. 3a). To confirm the presence of EV-like particles but also to assess the sample purity, EM analyses of VAT EV samples after 2 and 6 days from the initiation of culture were performed (Fig. 3b). Numerous spherical and cup-shaped structures were found in SEM and TEM micrographs, respectively. Micrographs also revealed the possible presence of impurities, including protein aggregates in 2-day samples. Therefore, we allocated 1-day and 2-day EV samples from both VAT and SAT cultures for apolipoprotein A1 (ApoA1) and calnexin WB (Fig. 3c). In these samples, the presence of ApoA1, a marker for HDL particles, was only detected in plasma, indicating that the EV fractions were not contaminated with blood-derived material. However, 1-day VAT EV sample was positive for calnexin, indicating that in the case of AT EV samples from ex vivo cultures, samples obtained after 2 days and onwards did not contain significant amounts of cell-derived contaminants. For further validations, 2-, 3-, and 6-day samples were pooled, and the presence of CD63, β-actin, and TSG101 was confirmed (Fig. 3d). Detailed validation was supplemented with high-resolution confocal microscopy analysis of 2-day EVs, further confirming the presence of CD9, CD63, and CD63-colocalizing FABP4 (Fig. 3e).

### VAT secretes more EVs than SAT and particle counts of these EVs positively correlate with plasma TAG levels

**Fig. 4:**
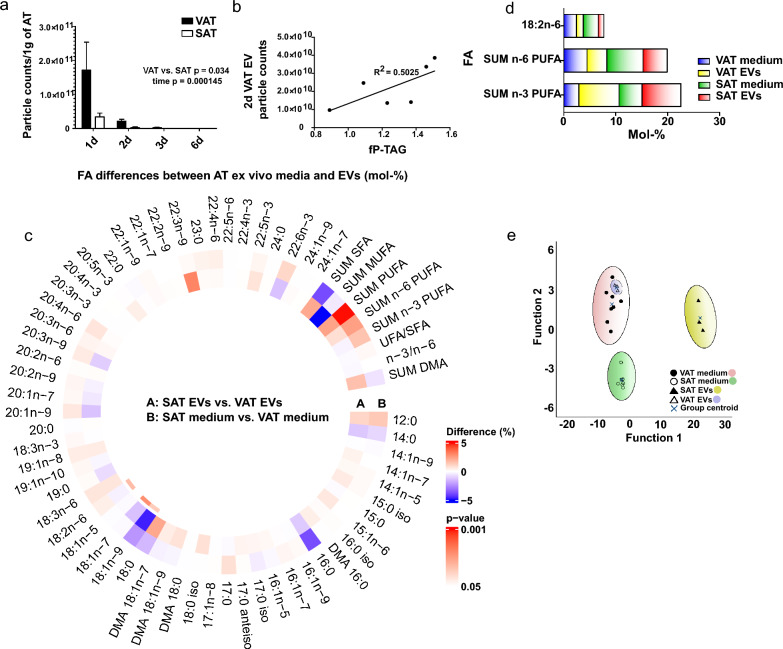
Comparison of the extracellular vesicles (EV) from visceral (VAT) and subcutaneous adipose tissue (SAT). Particle counts were obtained by nanoparticle tracking analysis (**a**). Generalized linear model VAT *vs*. SAT group *p* = 0.034, time *p* = 0.00145. Results include AT cultures of 6 patients, presented as mean + SEM. All EV counts have been normalized to 1 g of AT obtained for culturing. Correlation between 2d VAT EV particle counts with patient fasting plasma triacylglycerol levels (fP-TAG) (**b**). Spearman’s rank correlation efficient 0.829, *p* = 0.042. Fatty acid (FA) profiles of VAT and SAT EVs, as well as ex vivo culture media, were determined from total lipids with gas chromatography–mass spectrometry (**c**). Results are presented as percentage differences, calculated by subtracting the mol-% of each FA in the VAT group from the mol-% in the SAT group. Red indicates an increase in the SAT group, while blue indicates a decrease. FAs are listed in the order of increasing chromatographic retention time. *DMA* plasmalogen alkenyl chain-derived dimethyl acetal derivative, *SFA* saturated fatty acid, *MUFA* monounsaturated fatty acid, *PUFA* polyunsaturated fatty acid, unsaturated fatty acid (UFA) = MUFA + PUFA. *p ≤ 0.05 Mann–Whitney U test *vs*. VAT. Percentages of selected FAs in AT EVs and ex vivo culture media, presented as mean mol-% (**d**). Results of FA profiles were analyzed from 3 EV samples, which each corresponded to a pooled EV sample from 2 different patients. Media samples were obtained from 2 patients’ AT ex vivo cultures. The supervised discriminant analysis depicts the classification of FA signatures of VAT and SAT EVs as well as corresponding ex vivo media based on discriminant Functions 1 and 2 (**e**). Function 1 on the horizontal axis explained 89.3% of the variance, and Function 2 8.0% of the variance

To compare EV secretion between VAT and SAT, from which VAT is more strongly related to the metabolic complications of obesity, VAT and SAT samples of the same patients (n = 6) were cultured ex vivo. Particle concentrations were significantly higher in EV preparations obtained from VAT cultures than those from SAT cultures, suggesting that human VAT secretes more EVs than SAT when cultured ex vivo (Fig. 4a). Interestingly, 2-day VAT particle counts correlated with fasting plasma TAG levels of the patients (Fig. 4b). The most abundant FAs in AT EVs included PA (approximately 25% in both EV types), OA (18% and 13% in VAT and SAT EVs, respectively), SA (15% in both EV types), and DHA (5% in both EV types) (Supplementary Figure S7, see Additional File 1). The sum of all PUFAs was more than 14% in both EV types and n-3 PUFA levels were higher than n-6 PUFA sums. Furthermore, the FA compositions of EVs were compared to those of their respective culture media. While n-3 PUFA proportions tended to be higher in EVs compared to culture media, n-6 PUFAs were more abundant in culture media compared to EVs (Fig. 4d). LA proportions were lower in EVs than in culture media. The supervised DA of VAT and SAT EVs, as well as the corresponding culture media, showed that VAT and SAT media were separated from each other, indicating possible differences in FA uptake and/or secretion based on AT type (Fig. 4e). Also, interestingly, SAT EVs clearly segregated from SAT ex vivo medium, indicating specific incorporation of FAs into SAT EVs. However, VAT EV group overlapped with VAT ex vivo medium group. Function 1 separated VAT and SAT EV samples and explained 89.3% of the variance, with EPA having the most separating power. Function 2, separating VAT from SAT medium samples, explained 8.0% of the variance, and the FAs responsible for this were ARA, 12:0, and SA. Considering these FAs, EPA proportions tended to be higher in EV samples than in media samples, particularly in SAT EV samples (Fig. 4c; Supplementary Figure S7, see Additional File 1).

### Adipocyte- and patient AT-derived EVs carry TAGs, and may deliver excess lipid to circulation under reduced lipolytic conditions

**Fig. 5:**
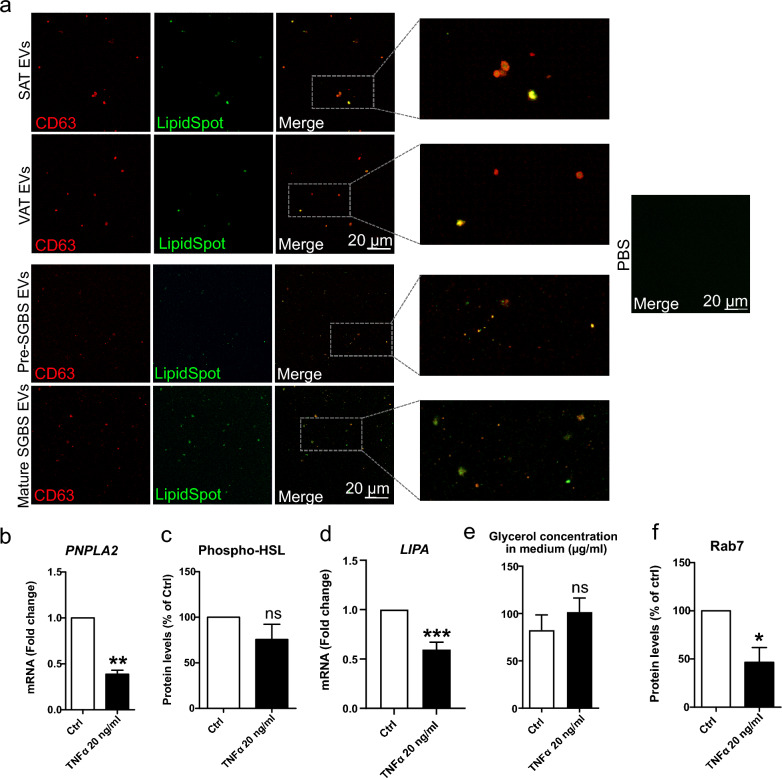
Studying the presence of lipids in adipose tissue (AT)-extracellular vesicles (EVs) by confocal microscopy (**a**). Confocal microscopy analysis was performed for Simpson Golabi Behmel Syndrome (SGBS) adipocyte and patient AT-derived EVs that were stained for lipids and CD63. EV samples from pre- and mature SGBS cells, as well as from patient visceral and subcutaneous adipose tissue (VAT and SAT, respectively) ex vivo cultures were stained with LipidSpot lipid droplet stain and fluorophore-conjugated CD63-antibody, after which samples were imaged with high-resolution confocal microscopy. PBS was included as non-EV control. The mRNA expression levels of *PNPLA2* in SGBS cells treated with 20 ng/ml TNFα for 24 h were analyzed by RT-qPCR (**b**). The values of six independent experiments are shown, presented as mean + SEM. **p = 0.002 (Mann–Whitney U test). Phosphorylated levels of hormone sensitive lipase (HSL) were studied by Western Blotting, from three independent experiments (**c**). Results are presented as mean + SEM. The mRNA expression levels of *LIPA* were studied by RT-qPCR, from nine independent experiments (**d**). Results are presented as mean + SEM, ***p = 0.0001 (Mann–Whitney U test). Glycerol concentration was determined from culture media of three experiments (**e**), and Rab7 protein levels from cells of four experiments (**f**). Results are presented as mean + SEM. *p = 0.014 (Mann–Whitney U test)

Based on biochemical analyses of mouse AT EVs, it was previously suggested that AT EVs contain TAGs, which raises an intriguing possibility for EV secretion being an additional mechanism for AT to relocate excess lipid and to communicate on the metabolic status of the body [40]. Therefore, we utilized high-resolution confocal microscopy of adipocyte and AT EV samples to investigate this possibility. EV samples from both pre- and mature SGBS cells, as well as from patient VAT and SAT ex vivo cultures were stained with LipidSpot stain and fluorophore-conjugated CD63-antibody, and then imaged with high-resolution confocal fluorescence microscopy (Fig. 5a). We observed particles in which CD63 and lipid droplet stain signals co-localized in all sample groups. In fact, some particles with the inner part of the EVs positive for LipidSpot signal, while having “CD63 cover” around, were also observed. These results indicate that AT EVs contain TAGs and that AT EV secretion may have a role in mobilizing and releasing lipid in addition to canonical lipolysis. To address this possibility, we then investigated further the interplay between increased EV secretion and canonical lipolysis in TNFα-treated SGBS adipocytes. Interestingly, the expression of *PNPLA2* and *LIPA*, encoding adipose tissue triglyceride lipase (ATGL) and lysosomal acid lipase (LAL), respectively, was strongly downregulated (Fig. 5b and d), and phosphorylated hormone sensitive lipase (HSL) protein levels showed a decreasing trend (Fig. 5c). However, despite these, glycerol concentration tended to increase in culture medium of cells (Fig. 5e), indicating that the breakdown of TAGs into free FAs and glycerol did occur, but not via canonical lipolysis, involving ATGL and HSL, and LAL in lysosomes. Also, the total levels of Rab7, the form of Rab GTPase that is present on lipid droplet surface and responsible for lipase-independent lipolysis via lipophagy, were decreased (Fig. 5f). Thus, our results reveal that TNFα-induced EV secretion occurs concomitantly with reduced lipolytic activity, suggesting that EV secretion could be promoted as a compensation to reduced lipolysis.

### Plasma EVs of patients with obesity contain more adiponectin-positive EVs than ones of normal-weight subjects

**Fig. 6:**
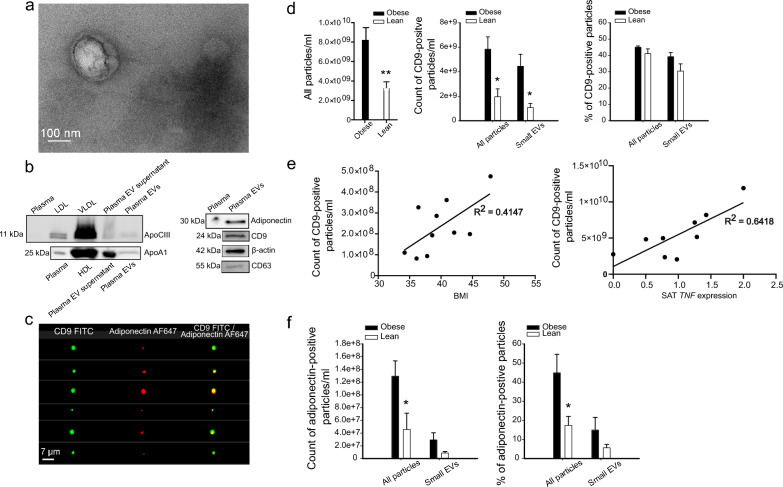
Amnis® ImageStream®X Mark II imaging flow cytometry analysis of plasma extracellular vesicles (EVs). Obese: Subjects with obesity, lean: Subjects with normal weight. Sample purity and the morphology of plasma EVs were first analyzed by transmission electron microscopy (**a**). Scale bar 100 nm. The presence of lipoprotein markers, apolipoprotein A1 (ApoA1) and apolipoprotein C-III (ApoCIII), as well as adiponectin, CD63, CD9, and β-actin EV markers, was analyzed by Western Blotting (**b**). Plasma EV samples were incubated with the mixture of CD9 and adiponectin antibodies, to test the double-positivity of adiponectin for CD9. Images of double-positive events are shown (**c**). All particles (objects/ml) and CD9-positive particles were measured, together with the percentage of CD9-positive particles (**d**) **p = 0.007 and *p = 0.022 (Mann–Whitney U test). Correlation between the number of CD9-positive EV particles with body mass index (BMI) and subcutaneous adipose tissue (SAT) *TNF* expression (**e**). CD9 particles and BMI: Spearman’s rank correlation coefficient 0.636, *p* = 0.048, CD9 particles and SAT *TNF* expression: Spearman’s rank correlation coefficient 0.733, *p* = 0.025. Adiponectin-positive particles were counted, and the percentage of adiponectin-positive particles from all particles was measured (**f**). EV samples from 6 normal-weight subjects and from 10 subjects with obesity were analyzed in total. *TNF* expression values were obtained from 9 subjects with obesity

To investigate the presence of AT-derived EVs in circulation, plasma EV samples labelled against CD9 and adiponectin were analyzed with Amnis® ImageStream®X Mark II imaging flow cytometer. Isolated plasma EVs were first characterized by TEM (Fig. 6a) and WB (Fig. 6b). WB revealed the presence of common EV markers, CD63, CD9, and β-actin, but also some HDL marker, ApoA1, and LDL/VLDL marker, ApoCIII, in plasma EVs. Importantly, adiponectin was also present, confirming its possible utility as AT EV marker in the following flow cytometry analyses. Three samples, two from subjects with obesity and one from a normal-weight subject were incubated with the mixture of CD9 and adiponectin antibodies (Fig. 6c). Most of the adiponectin positive events (≥ 95%) were double-positive for CD9, indicating that adiponectin staining reveals EV-resident adiponectin. The concentration of particles, obtained by side scatter, was higher in plasma EV samples from people with obesity compared to normal-weight controls (Fig. 6d). The number of CD9-positive events was also higher in plasma EV samples from people with obesity, but the percentage of CD9-positive particles of all particles was on the same level in both sample types. Interestingly, the number of CD9-positive particles of subjects with obesity positively correlated with BMI, as well as SAT *TNF* expression levels (Fig. 6e). Analyses of adiponectin-positive particles revealed that count and percentage of adiponectin-positive particles were higher in obese samples compared to lean samples, indicating higher AT EV secretion into circulation in obesity (Fig. 6f).

## Discussion

Growing evidence associates elevated circulating EV numbers with obesity, T2D, and non-alcoholic fatty liver disease in both human patients and rodent models [41, 42], although conflicting findings have also emerged [12]. Previous research on EV secretion from AT has yielded contrasting outcomes in murine adipocyte cell lines and AT depots, with some studies suggesting an increased secretion under obesity-related stimuli [4, 11, 22, 43, 44], and others indicating a decrease [45]. To address the knowledge gap in AT EV secretion in human obesity, we examined human SGBS adipocytes, as well as VAT and SAT samples from patients with obesity. As hypothesized, our findings indicate that inflammatory PA and TNFα promote EV secretion from SGBS adipocytes, and that patient VAT in ex vivo cultures secrete more EVs compared to SAT, providing novel insights into human AT EV secretion. We also explored changes in EV FA profiles under pro-inflammatory stimuli, suggesting EVs’ role as rapid responders to metabolic stress. Overall, we documented distinct FA profiles in human adipocyte EVs compared to previous murine data. Moreover, the positive correlation between VAT EV counts and plasma TAG levels, alongside the higher prevalence of adiponectin-positive EVs in the plasma of bariatric surgery patients compared to lean individuals gives further evidence for higher AT EV secretion in obesity. Importantly, increased EV counts in plasma were associated with high BMI and *TNF* expression in SAT. Finally, based on high-resolution confocal microscopy analysis, we show for the first time that AT and adipocyte EVs carry TAGs, and that EV secretion may act as a compensation to reduced lipolysis, suggesting a significant role of EVs in lipid mobilization. Our study increases our understanding of AT EVs’ roles in metabolic health and disorders in humans.

To our best knowledge, we provide here for the first time statistically significant data showing that human VAT secretes more EVs than the same amount of SAT, and the association between increased VAT EV counts with higher plasma TAG levels. In the experiments by [14], omental AT EV number correlated positively with HOMA-IR, but differences in EV secretion between omental AT and SAT were not observed. Previous data presented by [15] provided a hint of higher numbers of EVs secreted by VAT compared to SAT but, probably due to a small sample size, no robust interpretation could be made. Considering the increased EV secretion from SGBS adipocytes under inflammatory conditions, together with more inflammatory characteristics of VAT compared to SAT [46], it is plausible that inflammation is the driving force for the efficient EV secretion from VAT. The research in this particular field is in its early stages and, unlike in our study, proper EV validations with negative EV markers have rarely been included in previous studies utilizing AT EVs from ex vivo cultures. We do acknowledge that other factors, including differences in cellular composition and metabolic activity between VAT and SAT could affect EV secretion, independent of inflammation. More mechanistic studies investigating factors mediating EV secretion from AT are needed to confirm and better understand the observed differences in EV secretion between VAT and SAT.

Few previous attempts have been made to study if increased AT EV secretion due to obesity can be realized in vivo in circulation of mice and humans. Thomou et al. [10] observed that in mice, the majority of circulating EVs originated from AT, based on adipocyte-specific knockout mice of the miRNA-processing enzyme DICER, which resulted in decreased EV-resident miRNAs in circulation. However, Flaherty et al. [40] suggested in another mouse study that only a minority of circulating EVs originate from AT, but obesity increases EV secretion from AT. Camino et al. [15] proposed that plasma EVs positive for TGFB1 and mimecan, which are elevated in AT EVs, could be used as markers for tracking T2D and visceral obesity. However, the low tissue specificity of these proteins, particularly to AT, raises concerns about their reliability as AT EV markers, as TGFB1 is also expressed by immune cells and found in monocyte-derived EVs [47], and it has been associated with other inflammatory conditions and diseases, such as adenocarcinoma as well as pulmonary hypertension in HIV [48, 49]. To address this, we chose adiponectin, which is almost exclusively expressed in adipocytes and found in 3T3-L1, SGBSs- and human AT-derived EVs [14, 20], as a marker for AT-derived EVs in the flow cytometry analyses.

We established that the majority of adiponectin-positive EVs were also positive for the common EV marker CD9, indicating that EV-resident adiponectin was measured in our studies. We demonstrated that the percentage of adiponectin-positive EVs was higher in plasma EV samples of patients with obesity compared to normal-weight subjects, suggesting increased adipocyte EV secretion in human obesity. Interestingly, we found a positive association between increased CD9-positive plasma EV counts and BMIs as well as *TNF* expression in SAT, but not with glucose or insulin levels or with other patient parameters. Thus, our results support the association of increased EV counts with AT expansion and inflammation. This differs from previous studies that have shown correlations between plasma EV counts and glucose tolerance, IR, and HOMA-IR in people with obesity and patients with metabolic syndrome [21, 42]. It is noteworthy, as previously mentioned, that EV particle counts from VAT ex vivo cultures correlated with fasting plasma TAG levels. These results prompted the hypothesis, based on previous biochemical assays of mouse AT EVs [40], that EV secretion may be an additional mechanism for AT to redistribute excess TAGs, in addition to canonical lipolysis. To further support this hypothesis, we used high-resolution microscopy to study if TAGs were present in human SGBS and patient AT EVs and found co-localization of lipid stain with the common EV marker CD63. To our best knowledge, this is the first time when the presence of TAGs in EVs has been studied at the single EV level, providing more convincing evidence compared to biochemical assays. Indeed, a recent study with mouse material has shown similarities in obesity-related changes in FA profiles of VAT, VAT EVs, and plasma [22], suggesting that diet-induced changes in FA signatures of AT are reflected in AT EVs and, ultimately, in peripheral circulation, supporting the role of EVs as redistributors of AT TAGs, and FAs therein. In order to investigate further whether AT EVs could act in releasing TAGs into circulation, lipolysis pathways were investigated in more detail in TNFα-treated adipocytes, in which EV secretion was clearly demonstrated. Interestingly, our results tentatively suggest an increasing trend of glycerol levels in culture medium, despite the decreased *PNPLA2* and *LIPA* mRNA expression, as well as phospho-HSL protein levels. Although TNFα is commonly known as a factor stimulating lipolysis [50], differing evidence still exists. Apart from canonical ATGL/HSL mediated lipolysis, lipase-independent lipophagy, mediated by Rab7 may also contribute to the breakdown of TAGs. Intriguingly, protein levels of Rab7 were also downregulated, although this GTPase has also been implicated in exosome release [51]. To summarize, our data indicate that adipocyte EV secretion may be promoted as a compensation to reduced lipolysis, but further investigation is required to confirm this appealing possibility.

Previous research has primarily focused on proteomic and miRNA profiles of adipocyte EVs, largely overlooking their FA composition. Our study delved into EV secretion and FA signatures in human SGBS adipocytes. Differential FA profiles of pre- and mature SGBS cells, as well as cell and EV populations emerged, as indicated by our DAs. EVs from pre- and mature SGBS cells did not overlap, but were closely aligned, suggesting more profound differences between cells at different stages of development than in their EVs. Contrary to previous findings in murine 3T3-L1 cells [20], SGBS cells secreted more EVs after differentiation in our experiments, indicating that adipogenesis promotes EV secretion in human adipocytes. Thus, in addition to AT inflammation, increased adipogenesis in response to excess energy may also account for increased EV secretion from AT in obesity. Additionally, FA compositions diverged from previous 3T3-L1 data, with PA, SA, OA, and 14:0 being the most abundant FAs in SGBS EVs. In EVs from mature 3T3-L1 cells, LA has been reported to be the FA with the highest mol-%, after which PA, SA, and OA were the next most abundant FAs [20]. In our analyses, LA was only a minor FA in both SGBS and patient AT EVs. Additionally, PLA was present in EVs from both pre-SGBS and mature SGBS cells, while in 3T3-L1 EVs, it was only detected in pre-3T3-L1 cells [20]. Interestingly, OA was more abundant in mature SGBS cells compared to pre-SGBS cells, which was opposite to previously reported results in 3T3-L1 cells. Also, mature 3T3-L1 EVs had higher mol-% of OA compared to mature 3T3-L1 cells, while EVs from both pre- and mature SGBS cells had smaller OA proportions compared to the corresponding cells. Overall, our findings suggest selective differences in EV secretion and their FA signatures during the differentiation process between murine and human adipocytes.

Based on our DA data, human SGBS adipocyte EVs exhibited different FA profiles under TNFα, PA, and EPA treatments, stimuli relevant to nutrition, obesity-induced inflammation, or potential resolution promoted by EPA. Previous studies by Eguchi et al. [21] emphasized ARA, DHA, and EPA as major n-3 and n-6 PUFAs in EVs from PA-treated 3T3-L1 cells. High levels of ARA-containing lipid species were also documented in leptin-deficient mouse obesity models [22]. However, our research shows DPA, DHA, and LA among the most abundant PUFAs in PA-treated SGBS EVs. In fact, ARA was only a relatively minor FA in our SGBS and patient AT EVs and appeared to be more abundant in ex vivo culture medium than in the AT EVs. This contrasts with murine data, indicating unique human EV FA profiles under different stimuli. Notably, our results indicate stronger FA disparities in SGBS EVs *versus* cells during treatments, suggesting that metabolic stimuli-induced changes in FAs are detected in EVs earlier than in cells. EVs might function as swift responders to metabolic shifts, potentially conveying dietary FA changes across tissues. Elevated EV FA variances in obesity may mirror an AT attempt to counteract metabolic dysregulation. This could include the incorporation of SFAs into EVs for protective effects, alongside PLA from high ∆9-desaturation of PA, and SA and OA from PA elongation, mediating attenuated inflammatory signals and less lipotoxicity. Indeed, an increased proportion of SFAs compared to cells, a feature also reported elsewhere [52], was evident also in SGBS cells and EVs, suggesting that SFAs may be incorporated into EVs to alleviate harmful effects in AT. Also, despite the low PLA mol-% in EVs, the proportions of OA remained at a high level in EVs as in their parental cells, indicating the active incorporation of this ∆9-desaturation product into secreted EVs, as also observed with fibroblast-like synoviocytes [53]. Also, the proportion of OA was lower in EVs from EPA-treated cells compared to EVs from TNFα- and PA-treated cells, suggesting that in treatments with inflammatory components, ∆9-desaturation would have a protective role when producing OA to be incorporated into EVs instead of the SFAs.

There was a consistent abundance of PUFAs in both AT and SGBS EVs, surpassing culture medium and SGBS cells, respectively. Notably, beneficial n-3 PUFA proportions were as high as 7–8% in AT EVs and tended to be higher in EVs compared to culture media. For instance, DHA was more abundant in AT EVs compared to EVs from PA-, TNFα-, and EPA-treated SGBS cells. High proportions of n-3 PUFAs in adipocyte EVs may act as an adaptive response to metabolic stress. Due to substrate competition for enzymes, n-3 PUFAs reduce the production of inflammatory eicosanoids from n-6 PUFAs, and produce resolvins, protectins, and maresins having anti-inflammatory and pro-resolving activities [54, 55]. PUFAs can also enhance membrane fluidity, potentially influencing microenvironments and the activity of membrane-associated receptors and enzymes. DHA, for instance, has been linked to reduced Toll-like receptor 4 recruitment and subsequent, attenuated pro-inflammatory response [56]. Thus, EVs enriched in PUFAs, especially n-3 PUFAs, could serve as messengers of anti-inflammatory and beneficial cues to recipient cells, aiding resolution of inflammation.

Interestingly, according to our DA, SAT EV FA profiles were distinct from those of SAT ex vivo media, suggesting selective incorporation of FAs into SAT EVs rather than their release into culture medium. However, VAT EV group overlapped with VAT ex vivo medium, indicating that the overall differences in FA profiles between the two were not pronounced. A possible explanation for this could be the high lipolytic activity of VAT [46] leading to significant release of free FAs into the culture medium reducing the differences between the FA profiles of VAT EVs and medium. Second, the high similarity between VAT EVs and culture medium may simply be due to the high rate of EV secretion by VAT. The third possibility is that adipocytes in VAT more effectively recycle FAs from the culture medium into cells and, further, EVs. Indeed, exogenous FAs have been reported to modulate FA profiles of secreted EVs from bone marrow mesenchymal stem cells [57].

## Conclusions

In summary, our study reveals that factors associated with AT inflammation in obesity orchestrate significant changes in the secretion and FA profiles of AT EVs, indicating the role of AT EVs as rapid responders to metabolic stress. The detection of TAGs in adipocyte- and AT-EVs and high EV secretion despite signs of reduced lipolysis suggest a novel mechanism for redistribution of AT lipids. Coupled with the elevated prevalence of adiponectin-positive EVs in the plasma of subjects with obesity compared to lean individuals, this implies a crucial systemic function of AT EVs in TAG distribution. The findings deepen our understanding of AT biology and highlight the potential of EVs as prognostic and therapeutic targets for obesity and its associated conditions, while emphasizing the need for a deeper exploration of AT EV biological functions and clinical implications.

### Supplementary Information

Supplementary Material 1.

## Data Availability

All data generated or analysed during this study are included in this published article and its supplementary information files.

## Abbreviations

ALA: α-Linolenic acid (18:3n-3)
Alix: Programmed cell death 6 interacting protein
ANOVA: Analysis of variance
ApoA1: Apolipoprotein A1
ARA: Arachidonic acid (20:4n-6)
AT: Adipose tissue
BMI: Body mass index
CVA: *Cis*-vaccenic acid (18:1n-7)
DA: Discriminant analysis
DGLA: Dihomo-γ-linolenic acid (20:3n-6)
DHA: Docosahexaenoic acid (22:6n-3)
DMA: Dimethyl acetal derivative of alkenyl chain
DPA: Docosapentaenoic acid (22:5n-3)
EPA: Eicosapentaenoic acid (20:5n-3)
EV: Extracellular vesicle
FA: Fatty acid
FABP4: Fatty acid binding protein 4
FAME: Fatty acid methyl ester
GLA: γ-Linolenic acid (18:3n-6)
HDL: High-density lipoprotein
HOMA-IR: Homeostatic model assessment for insulin resistance
HSL: Hormone sensitive lipase
IR: Insulin resistance
LA: Linoleic acid (18:2n-6)
LDL: Low-density lipoprotein
MUFA: Monounsaturated fatty acid
NTA: Nanoparticle tracking analysis
OA: Oleic acid (18:1n-9)
PA: Palmitic acid (16:0)
PLA: Palmitoleic acid (16:1n-7)
PUFA: Polyunsaturated fatty acid
SA: Stearic acid (18:0)
SAT: Subcutaneous adipose tissue
SEM: Scanning electron microscopy
SFA: Saturated fatty acid
SGBS: Simpson Golabi Behmel Syndrome
T2D: Type 2 diabetes
TAG: Triacylglycerol
TEM: Transmission electron microscopy
TNFα: Tumor necrosis factor α
TSG101: Tumor susceptibility 101
VAT: Visceral adipose tissue
VLDL: Very-low-density lipoprotein
WB: Western Blotting
