# Supplementary material for: Increased secretion of adipocyte-derived extracellular vesicles is associated with adipose tissue inflammation and the mobilization of excess lipid in human obesity
Source: J Transl Med. 2024 Jul 4;22:623. doi: 10.1186/s12967-024-05249-w (PMC11225216; doi:10.1186/s12967-024-05249-w)
Supplement: Supplementary file 1 — Supplementary Material 1. [file 12967_2024_5249_MOESM1_ESM.pdf]

Supplementary Information, Additional file 1:

Table S1. Quantitative real-time PCR primer sequences.

**Gene**		Primer sequences	
C-C motif chemokine ligand 2	*CCL2*	Forward 5´	TCTCAAACTGAAGCTCGCAC
		Reverse 5´	GCATTGATTGCATCTGGCTGA
Interleukin-1α	*IL1A*	Forward 5´	CGCCAATGACTCAGAGGAAGA
		Reverse 5´	AGGGCGTCATTCAGGATGAA
Interleukin-1β	*IL1B*	Forward 5´	TGGAGCAACAAGTGGTGT
		Reverse 5´	TTGGGATCTACACTCTCCAGC
Interleukin-6	*IL6*	Forward 5´	TGCAATAACCACCCCTGACC
		Reverse 5´	GTGCCCATGCTACATTTGCC
C-X-C motif chemokine ligand 8	*CXCL8*	Forward 5´	CAGTGGACCACACTGCGCCAA
		Reverse 5´	TCCACAACCCTCTGCACCCAGTT
Hypoxanthine phosphoribosyltransferase 1	*HPRT1*	Forward 5´	CCTGGCGTCGTGATTAGTG
		Reverse 5´	ATCCAGCAGGTCAGCAAAGA
Patatin like phospholipase domain containing 2	*PNPLA2*	Forward 5´	CCGATGTTTCTGGAGTGCTG
		Reverse 5´	CACCAACAACACCGAGACC
Toll like receptor 4	*TLR4*	Forward 5´	CAGAGTTGCTTTCAATGGCATC
		Reverse 5´	AGACTGTAATCAAGAACCTGGAGG
Tumor necrosis factor	*TNF*	Forward 5´	CTGAACTTCGGGGTGATCG
		Reverse 5´	GCTTGGTGGTTTGCTACGAC
Lipase A, lysosomal acid type	*LIPA*	Forward 5´	GAAAATGCGGTTCTTGGGGT
		Reverse 5´	CAACTGGTTTGGGACCTTTGT

Table S2. Primary antibodies used in Western Blotting.

**Antibody**	**Cat. Number**	**Company**	**Dilution**
Adiponectin	sc-136131	Santa Cruz	1:200
Alix	92880	Cell Signaling Technology	1:1000
ApoA1	3350S	Cell Signaling Technology	1:1000
ApoCIII	ab31528	Abcam	1:1000
Mouse β-actin	A5441	Sigma-Aldrich	1:1000
Rabbit β-actin	A2066	Sigma-Aldrich	1:1000
Calnexin	2433	Cell Signaling Technology	1:1000
CD9	sc-59140	Santa Cruz	1:1000
CD63	ab59479	Abcam	1:1000
FABP4	AF3150	R&D systems	1:500
Phospho-HSL (Ser660)	45804	Cell Signaling Technology	1:1000
Rab7	9367	Cell Signaling Technology	1:1000
TSG101	ab125011	Abcam	1:1000

Table S3. Characteristics of normal-weight subjects from which fasting plasma samples were obtained (mean ± SEM).

Gender (male/female)	4/2
Age	37 ± 5.4
Body mass index (kg/m^2)	23.7 ± 0.8

FA	Pre-SGBS cells (Mol-%)	Mature SGBS cells (Mol-%)	Pre-SGBS EVs (Mol-%)	Mature SGBS EVs (Mol-%)	*P*
12:0	0.974 ± 0.719ab	0.158 ± 0.010a	0.657 ± 0.287b	0.970 ± 0.226b	0.030
14:0	1.909 ± 0.290a	2.014 ± 0.099a	3.778 ± 0.120b	3.946 ± 0.672b	0.005
14:1n-9	0.142 ± 0.083ab	0.019 ± 0.006a	0.189 ± 0.071b	0.233 ± 0.064b	0.010
14:1n-7	0.129 ± 0.056	0.035 ± 0.005	0.180 ± 0.074	0.189 ± 0.077	0.060
14:1n-5	0.437 ± 0.309	0.663 ± 0.035*	0.719 ± 0.284	1.041 ± 0.433	0.074
15:0 iso	0.264 ± 0.121b	0.013 ± 0.002a*	0.174 ± 0.079b	0.247 ± 0.114b	0.001
15:0	0.697 ± 0.382b	0.071 ± 0.010a*	0.261 ± 0.039b	0.322 ± 0.038b	0.001
15:1n-6	0.523 ± 0.223b	0.054 ± 0.012a*	0.470 ± 0.335b	0.611 ± 0.372b	0.016
16:0 iso	0.114 ± 0.008b	0.017 ± 0.003a*	0.193 ± 0.097b	0.267 ± 0.110b	0.001
DMA 16:0	1.151 ± 0.217	1.469 ± 0.305	0.521 ± 0.063	0.807 ± 0.286	0.298
16:0	18.539 ± 0.985a	27.959 ± 0.369b*	28.83 ± 0.700b	24.530 ± 3.701ab	0.005
16:1n-9	1.680 ± 0.296c	0.485 ± 0.027b*	0.182 ± 0.019a	0.343 ± 0.132ab	0.001
16:1n-7	2.401 ± 0.212a	21.296 ± 0.884b*	1.401 ± 0.049a	1.688 ± 0.067a*	0.000214
16:1n-5	0.373 ± 0.048b	0.049 ± 0.006a*	0.563 ± 0.113b	0.733 ± 0.285b	0.002
17:0 iso	0.395 ± 0.045b	0.051 ± 0.004a*	0.678 ± 0.253b	0.958 ± 0.231b	0.001
17:0 anteiso	0.568 ± 0.060b	0.050 ± 0.008a*	0.407 ± 0.123b	0.478 ± 0.092b	0.001
17:0	1.089 ± 0.362b	0.081 ± 0.013a*	1.073 ± 0.664b	0.807 ± 0.212b	0.001
17:1n-8	1.113 ± 0.211b	0.157 ± 0.017a*	0.280 ± 0.174a	0.309 ± 0.073ab	0.007
18:0 iso	0.322 ± 0.110b	0.037 ± 0.008a*	0.719 ± 0.149b	1.591 ± 0.735b	0.001
DMA 18:0	1.145 ± 0.267	0.560 ± 0.105	0.232 ± 0.061	0.356 ± 0.154	0.052
DMA 18:1n-9	0.384 ± 0.056	0.254 ± 0.051	0.330 ± 0.168	0.893 ± 0.642	0.373
DMA 18:1n-7	0.426 ± 0.076	0.623 ± 0.129	1.047 ± 0.342	0.787 ± 0.309	0.420
18:0	13.170 ± 1.520b	3.814 ± 0.321a*	18.493 ± 1.819b	13.843 ± 3.034b	0.001
18:1n-9	25.646 ± 2.540ab	29.663 ± 1.475b	21.912 ± 2.294a	20.497 ± 3.033a	0.021
18:1n-7	4.857 ± 0.634a	6.621 ± 0.302b*	2.592 ± 0.312a	2.337 ± 0.447a	0.001
18:1n-5	0.427 ± 0.089	0.507 ± 0.018*	0.438 ± 0.087	0.425 ± 0.100	0.160
18:2n-6	2.757 ± 0.422b	0.273 ± 0.055a*	1.182 ± 0.028b	1.262 ± 0.143b	0.000221
18:3n-6	0.100 ± 0.010ab	0.025 ± 0.015a*	0.329 ± 0.108b	0.222 ± 0.094b	0.002
19:0	0.175 ± 0.092b	0.016 ± 0.006a*	0.481 ± 0.251b	2.183 ± 1.166b	0.001
19:1n-10	0.171 ± 0.047b	0.024 ± 0.006a*	0.418 ± 0.162b	1.001 ± 0.567b	0.001
19:1n-8	0.174 ± 0.017b	0.037 ± 0.012a*	0.678 ± 0.385b	0.748 ± 0.396b	0.002
18:3n-3	0.167 ± 0.061b	0.020 ± 0.007a*	0.336 ± 0.130b	0.886 ± 0.409b	0.001
20:0	0.222 ± 0.027b	0.058 ± 0.004a*	0.164 ± 0.033b	0.178 ± 0.043b	0.001
20:1n-9	0.258 ± 0.038	0.236 ± 0.028	0.296 ± 0.102	1.428 ± 1.082	0.866
20:1n-7	0.237 ± 0.054	0.165 ± 0.019	0.249 ± 0.100	0.321 ± 0.131	0.816
20:2n-9	0.370 ± 0.144	0.085 ± 0.009*	0.470 ± 0.429	0.478 ± 0.196	0.061
20:2n-6	0.092 ± 0.012b	0.021 ± 0.008a*	0.092 ± 0.061ab	0.074 ± 0.029ab	0.028
20:3n-9	0.296 ± 0.133a	0.652 ± 0.085b*	0.136 ± 0.037a	0.593 ± 0.462ab	0.022
20:3n-6	1.278 ± 0.131b	0.061 ± 0.012a*	0.444 ± 0.165ab	0.861 ± 0.367ab	0.003
20:4n-6	3.756 ± 0.657b	0.389 ± 0.057a*	0.370 ± 0.072a	0.464 ± 0.141a	0.004
20:3n-3	0.113 ± 0.085b	0.024 ± 0.018a*	0.034 ± 0.007b	0.040 ± 0.013b	0.038
20:4n-3	0.068 ± 0.023b	0.009 ± 0.002a*	0.034 ± 0.009b	0.058 ± 0.018b	0.008
20:5n-3	0.496 ± 0.097b	0.075 ± 0.015a*	0.667 ± 0.317b	0.311 ± 0.115b	0.003
22:0	0.295 ± 0.066b	0.076 ± 0.008a*	0.203 ± 0.094ab	0.150 ± 0.031ab	0.011
22:1n-9	0.056 ± 0.019ab	0.026 ± 0.006a	0.183 ± 0.110b	0.130 ± 0.048b	0.031
22:1n-7	0.024 ± 0.007	0.030 ± 0.005	0.062 ± 0.017	0.054 ± 0.025	0.308
22:2n-9	0.029 ± 0.008ab	0.013 ± 0.003a	0.052 ± 0.008b	0.035 ± 0.01ab	0.017
22:3n-9	0.544 ± 0.285ab	0.099 ± 0.036a	2.261 ± 0.760b	2.506 ± 1.837b	0.004
23:0	0.226 ± 0.149	0.218 ± 0.049	0.418 ± 0.338	0.131 ± 0.063	0.740
22:4n-6	0.811 ± 0.127b	0.098 ± 0.016a*	0.166 ± 0.129a	0.164 ± 0.113a	0.006
22:5n-6	0.147 ± 0.018b	0.015 ± 0.002a*	0.130± 0.047b	0.143 ± 0.060b	0.001
22:4n-3	0.034 ± 0.012b	0.005 ± 0.001a*	0.021 ± 0.005b	0.038 ± 0.020b	0.001
22:5n-3	2.047 ± 0.414b	0.138 ± 0.022a*	0.679 ± 0.132b	1.435 ± 0.711b	0.002
24:0	0.779 ± 0.143b	0.095 ± 0.010a*	0.109 ± 0.017a	0.195 ± 0.074ab	0.003
22:6n-3	4.926 ± 1.690b	0.125 ± 0.026a*	2.920 ± 2.165b	4.535 ± 2.740b	0.001
24:1n-9	0.410 ± 0.067b	0.164 ± 0.024a*	0.073 ± 0.010a	0.089 ± 0.032a	0.003
24:1n-7	0.063 ± 0.013b	0.011 ± 0.002a*	0.022 ± 0.005ab	0.077 ± 0.039b	0.007
SUM SFA	39.740 ± 1.012b	34.726 ± 0.353a*	56.640 ± 0.796b	50.799 ± 4.748b	0.0000296
SUM MUFA	39.122 ± 2.084a	60.242 ± 0.917b*	30.907 ± 1.890a	32.253 ± 2.069a	0.000244
SUM PUFA	18.032 ± 1.975b	2.126 ± 0.236a*	10.322 ± 2.736b	14.105 ± 4.532b	0.001
SUM n-6 PUFA	8.942 ± 0.668b	0.882 ± 0.102a*	2.714 ± 0.229b	3.190 ± 0.575b	0.000221
SUM n-3 PUFA	7.851 ± 1.810b	0.396 ± 0.057a*	4.690 ± 2.227b	7.304 ± 3.494b	0.002
UFA/SFA	1.447 ± 0.068a	1.799 ± 0.036b*	0.729 ± 0.028a	0.958 ± 0.165a	0.000262
n-3/n-6 PUFA	0.929 ± 0.280b	0.451 ± 0.050a*	1.610 ± 0.634b	1.952 ± 0.783b	0.011
SUM DMA	3.106 ± 0.493	2.906 ± 0.585	2.131 ± 0.585	2.844 ± 1.089	0.825

0 % 100

Figure S1. Fatty acid (FA) profiles of pre- and mature ~~SGBS~~ cells as well as of their extracellular vesicles (EVs). Detailed FAs were determined from total lipids with gas chromatography–mass spectrometry. Results are presented as mol-%, mean ± SEM. FAs are listed in the order of increasing chromatographic retention time. DMA = plasmalogen alkenyl chain-derived dimethyl acetal derivative, SFA = saturated fatty acid, MUFA = monounsaturated fatty acid, PUFA = polyunsaturated fatty acid, UFA = MUFA + PUFA. Means with no common letter are significantly different from each

other within a row (Kruskal–Wallis ANOVA). * $p \leq 0.05$ Mann–Whitney U test *vs*. premature cells or EVs. Results of EV samples were obtained from 3, and results of cell samples from 6 independently obtained samples.

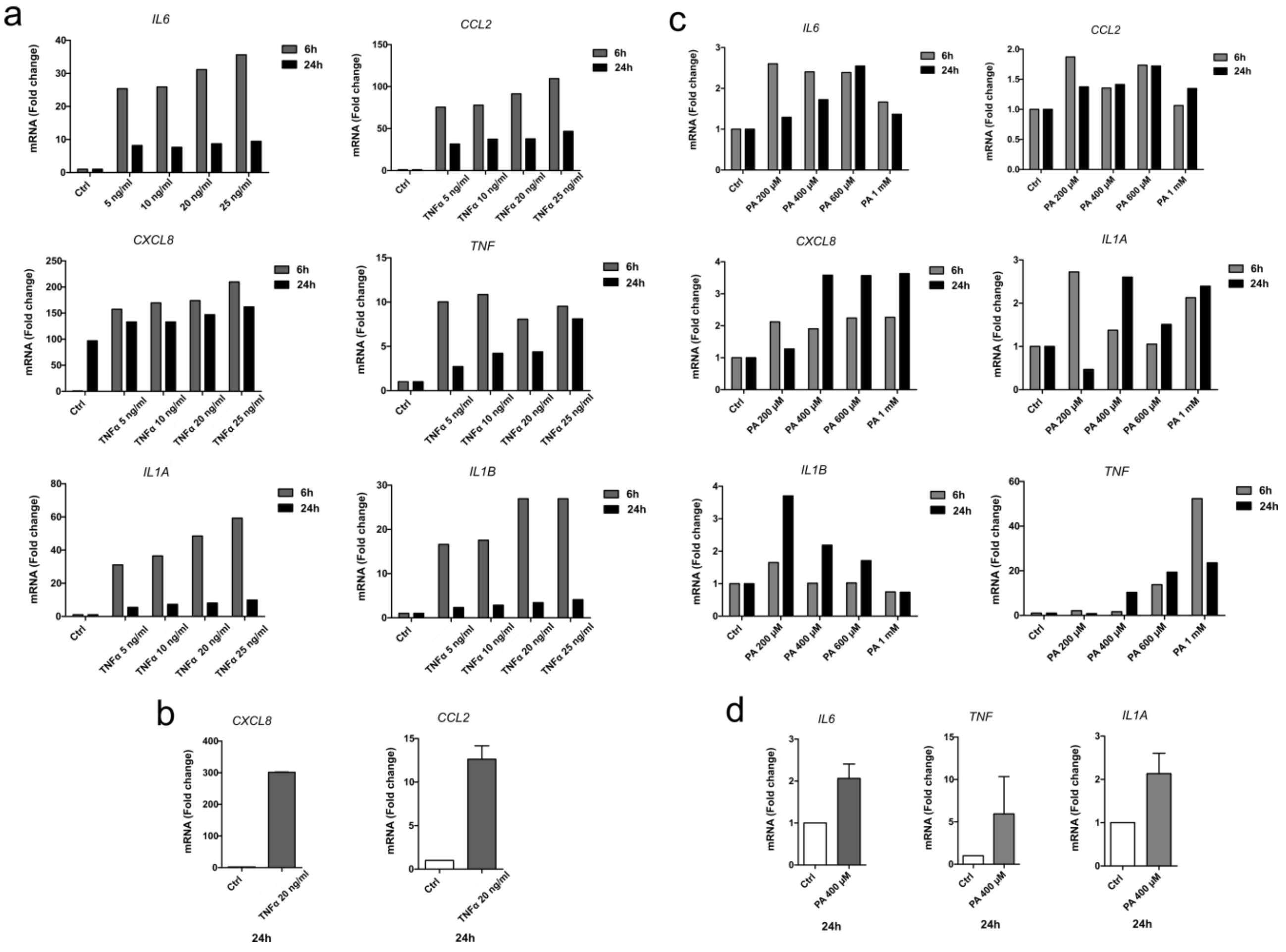

Figure S2. Responses of Simpson Golabi Behmel Syndrome ~~(SGBS)~~ cells to tumor necrosis factor α (TNFα) and palmitic acid (PA, 16:0) treatments. The mRNA expression levels of inflammation-related genes, including *IL6*, *CCL2*, *CXCL8*, *TNF*, *IL1A*, and *IL1B* after 6 h and 24 h TNFα treatments were analyzed by RT-qPCR (a). The values of one experiment are shown. Based on these results, final concentration of 20 ng/ml of TNFα was chosen and *CXCL8* and *CCL2* mRNA levels were further analyzed from two independent experiments (means + SEM) (b). The mRNA expression levels of inflammation-related genes, including *IL6*, *CCL2*, *CXCL8*, *IL1A*, *IL1B*, and *TNF* after 6 h and 24 h PA treatments were analyzed from one experiment (c). Based on these results, final concentration of 400 µM of PA was chosen and *IL6*, *TNF*, and *IL1A* mRNA levels were further analyzed from two independent experiments (means + SEM) (d).

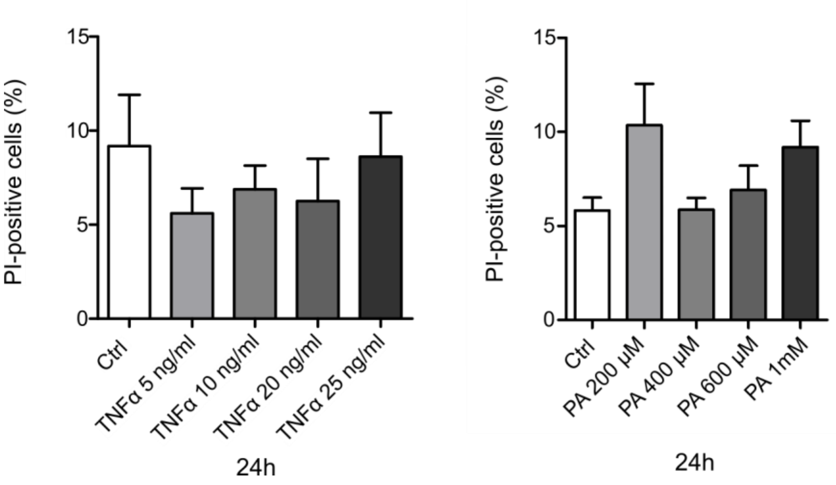

Figure S3. Viability and responses of Simpson Golabi Behmel Syndrome (SGBS) cells to tumor necrosis factor α (TNFα) and palmitic acid (PA, 16:0) treatments. SGBS cells were treated with different concentrations of TNFα and PA for 24h, and cell viability was evaluated by propidium iodide (PI) staining and confocal microscopy. PI-positive cells were manually counted from one experiment, from 10 images per each sample group. Results are presented as mean % of PI-positive cells from total cell count + SEM.

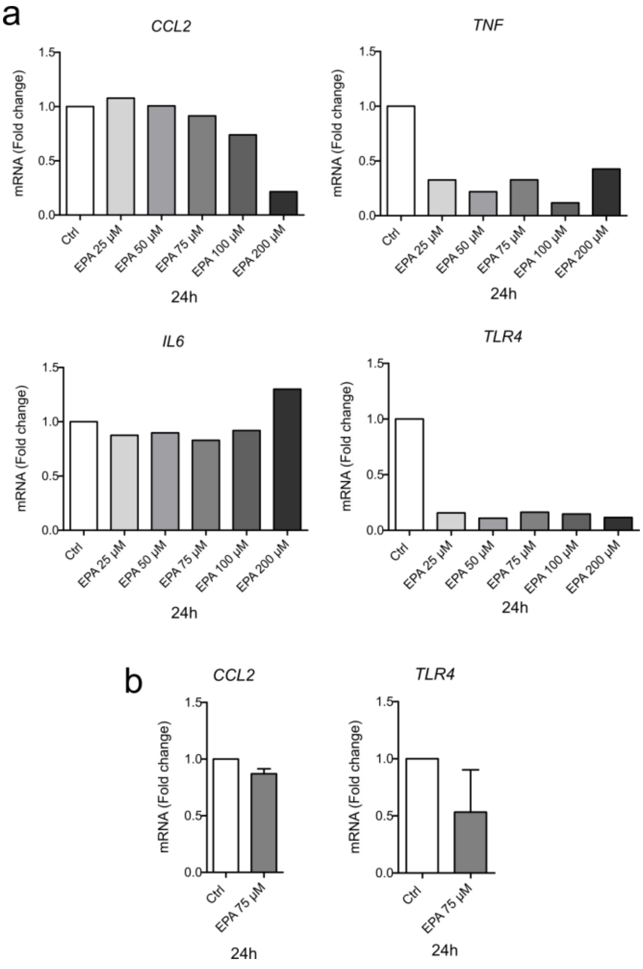

Figure S4. Responses of Simpson Golabi Behmel Syndrome ~~(SGBS)~~ cells to 24-h eicosapentaenoic acid (EPA, 20:5n-3) treatments. The mRNA expression levels of inflammation-related genes, including *CCL2*, *TNF*, *IL6*, and *TLR4* were analyzed by RT-qPCR from one experiment (a). Based on these results, final concentration of 75 µM of EPA was chosen, and *CCL2* and *TLR4* mRNA levels were further analyzed from two independent experiments (b). The means + SEM are shown.

FA	SGBS ctrl cells (Mol-%)	TNFα cells (Mol-%)	TNFα ctrl EVs (Mol-%)	TNFα EVs (Mol-%)	P
12:0	0.220 ± 0.036	0.302 ± 0.068	0.744 ± 0.301	0.562 ± 0.151	0.241
14:0	2.645 ± 0.261a	2.857 ± 0.124ab	4.250 ± 0.805bc	4.809 ± 0.804c	0.035
14:1n-9	0.014 ± 0.003a	0.012 ± 0.004a	0.312 ± 0.103b	0.111 ± 0.057ab	0.014
14:1n-7	0.037 ± 0.004	0.044 ± 0.007	0.175 ± 0.077	0.159 ± 0.118	0.079
14:1n-5	1.323 ± 0.185	1.569 ± 0.189	1.526 ± 0.615	0.748 ± 0.313	0.350
15:0 iso	0.028 ± 0.008a	0.035 ± 0.015a	0.200 ± 0.088b	0.151 ± 0.065b	0.017
15:0	0.090 ± 0.005a	0.115 ± 0.027a	0.359 ± 0.064b	0.293 ± 0.027b	0.009
15:1n-6	0.022 ± 0.006a	0.025 ± 0.008a	0.625 ± 0.261b	0.397 ± 0.125b	0.009
16:0 iso	0.020 ± 0.005a	0.016 ± 0.004a	0.397 ± 0.169b	0.215 ± 0.042b	0.009
DMA 16:0	0.315 ± 0.071	0.207 ± 0.014	0.487 ± 0.155	0.614 ± 0.226	0.162
16:0	27.237 ± 0.419	26.802 ± 0.623	27.905 ± 1.589	29.317 ± 4.166	0.633
16:1n-9	0.407 ± 0.033b	0.479 ± 0.103b	0.275 ± 0.041ab	0.144 ± 0.025a*	0.022
16:1n-7	33.684 ± 1.915b	34.392 ± 1.170b	2.019 ± 0.546a	1.562 ± 0.060a	0.010
16:1n-5	0.055 ± 0.009a	0.064 ± 0.015a	0.896 ± 0.099b	0.707 ± 0.300b	0.013
17:0 iso	0.059 ± 0.010a	0.062 ± 0.006a	0.608 ± 0.219b	0.410 ± 0.096b	0.010
17:0 anteiso	0.031 ± 0.003ab	0.025 ± 0.003a	0.360 ± 0.068c	0.322 ± 0.040bc	0.006
17:0	0.051 ± 0.005a	0.120 ± 0.070a	2.101 ± 1.080b	1.201 ± 0.277b	0.013
17:1n-8	0.170 ± 0.013	0.163 ± 0.012	0.200 ± 0.034	0.161 ± 0.013	0.702
18:0 iso	0.033 ± 0.007a	0.025 ± 0.004a	1.397 ± 0.672b	0.820 ± 0.223b	0.009
DMA 18:0	0.097 ± 0.017	0.083 ± 0.009	0.278 ± 0.093	0.218 ± 0.085	0.131
DMA 18:1n-9	0.054 ± 0.007ab	0.043 ± 0.005a	0.306 ± 0.080b	0.225 ± 0.124b	0.025
DMA 18:1n-7	0.119 ± 0.021ab	0.083 ± 0.009a	1.420 ± 0.568c	1.150 ± 0.418bc	0.006
18:0	1.502 ± 0.162a	1.447 ± 0.186a	16.247 ± 2.450b	17.256 ± 2.738b	0.010
18:1n-9	20.535 ± 2.136	19.911 ± 1.289	20.308 ± 2.454	21.232 ± 2.343	0.960
18:1n-7	8.257 ± 0.377b	8.096 ± 0.428b	2.949 ± 0.262a	2.708 ± 0.213a	0.009
18:1n-5	0.527 ± 0.048	0.518 ± 0.022	0.374 ± 0.100	0.579 ± 0.192	0.470
18:2n-6	0.551 ± 0.021a	0.575 ± 0.017a	1.267 ± 0.133b	1.112 ± 0.229b	0.009
18:3n-6	0.011 ± 0.002ab	0.005 ± 0.001a*	0.190 ± 0.055b	0.237 ± 0.119b	0.004
19:0	0.016 ± 0.003ab	0.008 ± 0.003a	0.854 ± 0.487c	0.544 ± 0.344bc	0.004
19:1n-10	0.022 ± 0.005ab	0.014 ± 0.004a	0.388 ± 0.124bc	0.551 ± 0.233c	0.006
19:1n-8	0.024 ± 0.005a	0.028 ± 0.008a	0.502 ± 0.234b	0.928 ± 0.781b	0.012
18:3n-3	0.019 ± 0.004ab	0.011 ± 0.003a	0.343 ± 0.091bc	0.621 ± 0.369c	0.006
20:0	0.049 ± 0.008ab	0.042 ± 0.005a	0.136 ± 0.044bc	0.278 ± 0.033c*	0.009
20:1n-9	0.163 ± 0.020	0.166 ± 0.016	0.834 ± 0.383	1.357 ± 1.215	0.137
20:1n-7	0.149 ± 0.011	0.148 ± 0.015	0.279 ± 0.031	0.349 ± 0.227	0.142
20:2n-9	0.054 ± 0.008	0.046 ± 0.007	0.340 ± 0.256	0.669 ± 0.635	0.303
20:2n-6	0.010 ± 0.003ab	0.005 ± 0.001a	0.051 ± 0.009c	0.055 ± 0.029bc	0.012
20:3n-9	0.395 ± 0.036	0.395 ± 0.046	0.440 ± 0.036	1.267 ± 0.610	0.631
20:3n-6	0.023 ± 0.003a	0.025 ± 0.005a	0.548 ± 0.148b	0.486 ± 0.356b	0.008
20:4n-6	0.231 ± 0.030	0.203 ± 0.032	0.430 ± 0.066	0.375 ± 0.251	0.214
20:3n-3	0.006 ± 0.002ab	0.005 ± 0.002a	0.045 ± 0.013c	0.044 ± 0.021bc	0.015
20:4n-3	0.006 ± 0.002a	0.004 ± 0.001a	0.055 ± 0.019b	0.057 ± 0.035b	0.008
20:5n-3	0.054 ± 0.023ab	0.040 ± 0.011a	0.251 ± 0.132b	0.187 ± 0.09b	0.034
22:0	0.043 ± 0.010a	0.043 ± 0.013a	0.170 ± 0.07b	0.115 ± 0.043ab	0.045
22:1n-9	0.019 ± 0.002a	0.016 ± 0.002a	0.043 ± 0.006b	0.025 ± 0.005ab*	0.022
22:1n-7	0.009 ± 0.001a	0.009 ± 0.002a	0.039 ± 0.003b	0.032 ± 0.008b	0.009
22:2n-9	0.005 ± < 0.001	0.004 ± 0.001a	0.040 ± 0.002c	0.018 ± 0.006bc*	0.007
22:3n-9	0.063 ± 0.023a	0.041 ± 0.022a	1.642 ± 0.559b	0.554 ± 0.395b	0.009
23:0	0.037 ± 0.013	0.034 ± 0.007	0.158 ± 0.043	0.486 ± 0.457	0.071
22:4n-6	0.089 ± 0.012	0.079 ± 0.012	0.081 ± 0.019	0.145 ± 0.123	0.781
22:5n-6	0.020 ± 0.005a	0.013 ± 0.002a	0.146 ± 0.073b	0.150 ± 0.061b	0.008
22:4n-3	0.007 ± 0.002	0.009 ± 0.002	0.031 ± 0.003	0.024 ± 0.011	0.055
22:5n-3	0.127 ± 0.015a	0.107 ± 0.021a	1.390 ± 0.644b	0.899 ± 0.242b	0.009
24:0	0.068 ± 0.023	0.060 ± 0.011	0.106 ± 0.010	0.114 ± 0.049	0.337
22:6n-3	0.142 ± 0.024a	0.319 ± 0.142a	2.321 ± 1.316b	2.150 ± 1.026b	0.015
24:1n-9	0.048 ± 0.007	0.044 ± 0.015	0.094 ± 0.012	0.083 ± 0.025	0.084
24:1n-7	0.009 ± 0.002a	0.006 ± 0.001a	0.066 ± 0.024b	0.014 ± 0.006ab*	0.037
SUM SFA	32.127 ± 0.526a	31.993 ± 0.564a	55.992 ± 0.824b	56.894 ± 5.479b	0.010
SUM MUFA	65.474 ± 0.637b	65.706 ± 0.598b	31.903 ± 1.153a	31.849 ± < 0.001	0.010
SUM PUFA	1.814 ± 0.103a	1.885 ± 0.249a	9.613 ± 1.316b	9.050 ± 5.479b	0.010
SUM n-6 PUFA	0.936 ± 0.049a	0.904 ± 0.052a	2.713 ± 0.067b	2.561 ± 1.135b	0.009
SUM n-3 PUFA	0.362 ± 0.053a	0.495 ± 0.153a	4.437 ± 0.957b	3.981 ± 3.806b	0.009
UFA/SFA	2.099 ± 0.054b	2.118 ± 0.055b	0.742 ± 0.012a	0.749 ± 0.679a	0.009
n-3/n-6 PUFA	0.382 ± 0.046a	0.526 ± 0.144ab	1.638 ± 0.365c	1.417 ± 1.674bc	0.015
SUM DMA	0.585 ± 0.114ab	0.415 ± 0.031a	2.492 ± 0.809c	2.207 ± 0.157bc	0.014

0 % 100

FA	Ctrl cells (Mol-%)	PA cells (Mol-%)	Ctrl EVs (Mol-%)	PA EVs (Mol-%)	P
12:0	0.259 ± 0.050a	0.218 ± 0.021a	0.507 ± 0.064ab	1.130 ± 0.43b*	0.011
14:0	3.103 ± 0.330a	2.787 ± 0.229a	5.111 ± 0.549b	4.181 ± 0.156ab*	0.130
14:1n-9	0.012 ± 0.003a	0.011 ± 0.001a	0.237 ± 0.037b	0.187 ± 0.024b	0.008
14:1n-7	0.074 ± 0.023ab	0.036 ± 0.004a	0.229 ± 0.097b	0.227 ± 0.083b	0.016
14:1n-5	1.645 ± 0.300c	1.182 ± 0.144bc	0.643 ± 0.092a	0.662 ± 0.166ab	0.019
15:0 iso	0.043 ± 0.016ab	0.015 ± 0.002a	0.440 ± 0.271c	0.111 ± 0.019bc	0.008
15:0	0.101 ± 0.012a	0.098 ± 0.012a	0.355 ± 0.083b	0.252 ± 0.007b	0.008
15:1n-6	0.050 ± 0.015a	0.024 ± 0.006a	0.557 ± 0.330b	0.282 ± 0.066b	0.008
16:0 iso	0.020 ± 0.005a	0.014 ± 0.001a	0.327 ± 0.089b	0.293 ± 0.027b	0.008
DMA 16:0	0.195 ± 0.016a	0.350 ± 0.129ab	0.559 ± 0.267b	0.608 ± 0.185b	0.045
16:0	26.293 ± 0.88a	31.055 ± 0.538b*	28.551 ± 1.852ab	27.636 ± 2.242ab	0.035
16:1n-9	0.746 ± 0.189b	0.872 ± 0.364b	0.264 ± 0.098ab	0.115 ± 0.012a	0.016
16:1n-7	35.551 ± 2.763b	30.958 ± 1.823b	1.378 ± 0.168a	1.255 ± 0.481a	0.006
16:1n-5	0.039 ± 0.003a	0.047 ± 0.005ab	0.561 ± 0.065c	0.453 ± 0.084bc	0.006
17:0 iso	0.061 ± 0.008	0.052 ± 0.007	0.768 ± 0.342	0.861 ± 0.410	0.051
17:0 anteiso	0.042 ± 0.011a	0.034 ± 0.004a	0.336 ± 0.044b	0.425 ± 0.196b	0.008
17:0	0.043 ± 0.006a	0.048 ± 0.006a	1.503 ± 1.038b	1.625 ± 1.136b	0.008
17:1n-8	0.191 ± 0.022	0.159 ± 0.012	0.184 ± 0.039	0.658 ± 0.441	0.173
18:0 iso	0.035 ± 0.007a	0.022 ± 0.004a	0.938 ± 0.542b	1.269 ± 0.065b	0.006
DMA 18:0	0.066 ± 0.009	0.127 ± 0.054	0.227 ± 0.040	0.242 ± 0.100	0.132
DMA 18:1n-9	0.037 ± 0.002a	0.063 ± 0.021ab	0.192 ± 0.064bc	0.383 ± 0.045c	0.008
DMA 18:1n-7	0.084 ± 0.007a	0.128 ± 0.046ab	1.118 ± 0.599bc	1.961 ± 0.643c	0.012
18:0	1.215 ± 0.212a	1.827 ± 0.345ab	19.535 ± 1.481c	24.244 ± 8.197bc	0.005
18:1n-9	19.130 ± 2.537	18.801 ± 2.105	20.619 ± 1.707	16.793 ± 6.043	0.892
18:1n-7	7.984 ± 0.263bc	8.595 ± 0.452c	3.457 ± 0.778ab	1.453 ± 0.038a*	0.005
18:1n-5	0.871 ± 0.275	0.507 ± 0.032*	0.443 ± 0.060	0.439 ± 0.151	0.104
18:2n-6	0.106 ± 0.011	0.109 ± 0.012	1.390 ± 0.215	0.873 ± 0.410	0.072
18:3n-6	0.012 ± 0.003a	0.010 ± 0.003a	0.240 ± 0.089b	0.457 ± 0.089b	0.007
19:0	0.006 ± 0.001a	0.009 ± 0.002ab	0.264 ± 0.151c	0.274 ± 0.151bc	0.005
19:1n-10	0.015 ± 0.004a	0.018 ± 0.007a	0.430 ± 0.085b	0.673 ± 0.182b	0.008
19:1n-8	0.028 ± 0.003a	0.032 ± 0.010a	0.278 ± 0.030b	0.703 ± 0.471b	0.008
18:3n-3	0.010 ± 0.003a	0.010 ± 0.003a	1.287 ± 0.657b	0.510 ± 0.255b	0.008
20:0	0.034 ± 0.002a	0.043 ± 0.007a	0.178 ± 0.041b	0.355 ± 0.183b	0.007
20:1n-9	0.153 ± 0.014a	0.181 ± 0.016ab	0.287 ± 0.030b	0.960 ± 0.772ab	0.042
20:1n-7	0.117 ± 0.011	0.146 ± 0.013	0.381 ± 0.289	0.350 ± 0.21	0.502
20:2n-9	0.058 ± 0.008a	0.055 ± 0.009a	0.173 ± 0.073b	0.943 ± 0.658b	0.014
20:2n-6	0.014 ± 0.004ab	0.006 ± 0.001a	0.103 ± 0.048bc	0.117 ± 0.065c	0.007
20:3n-9	0.323 ± 0.038	0.363 ± 0.079	1.266 ± 1.173	1.540 ± 1.313	0.844
20:3n-6	0.025 ± 0.004a	0.026 ± 0.005a	0.208 ± 0.075b	0.252 ± 0.137b	0.011
20:4n-6	0.226 ± 0.030	0.247 ± 0.048	0.360 ± 0.095	0.330 ± 0.049	0.291
20:3n-3	0.006 ± 0.002a	0.004 ± 0.001a	0.038 ± 0.012b	0.050 ± 0.017b	0.010
20:4n-3	0.007 ± 0.002a	0.007 ± 0.001a	0.074 ± 0.025b	0.036 ± 0.009b	0.007
20:5n-3	0.051 ± 0.010a	0.046 ± 0.016a	0.198 ± 0.087ab	0.352 ± 0.042b	0.040
22:0	0.024 ± 0.005a	0.034 ± 0.008ab	0.185 ± 0.050bc	0.301 ± 0.082c	0.006
22:1n-9	0.020 ± 0.004	0.021 ± 0.004	0.197 ± 0.165	0.113 ± 0.045	0.146
22:1n-7	0.009 ± 0.002a	0.009 ± 0.001ab	0.040 ± 0.015bc	0.045 ± 0.017c	0.017
22:2n-9	0.008 ± 0.003a	0.005 ± 0.001a	0.021 ± 0.002b	0.027 ± 0.009b	0.024
22:3n-9	0.165 ± 0.043ab	0.030 ± 0.01a*	1.214 ± 0.512b	0.722 ± 0.236b	0.003
23:0	0.214 ± 0.118	0.119 ± 0.042	0.122 ± 0.050	0.069 ± 0.032	0.838
22:4n-6	0.082 ± 0.012	0.086 ± 0.028	0.088 ± 0.032	0.126 ± 0.022	0.395
22:5n-6	0.020 ± 0.004a	0.016 ± 0.004a	0.096 ± 0.042ab	0.268 ± 0.066b*	0.023
22:4n-3	0.006 ± 0.002	0.007 ± 0.002	0.022 ± 0.009	0.035 ± 0.015	0.057
22:5n-3	0.112 ± 0.021ab	0.093 ± 0.032a	1.119 ± 0.605b	1.027 ± 0.434b	0.035
24:0	0.050 ± 0.005	0.036 ± 0.008	0.108 ± 0.037	0.105 ± 0.031	0.066
22:6n-3	0.158 ± 0.035a	0.155 ± 0.041a	0.419 ± 0.041b	0.554 ± 0.089b	0.008
24:1n-9	0.045 ± 0.007	0.040 ± 0.011	0.152 ± 0.060	0.063 ± 0.018	0.408
24:1n-7	0.007 ± 0.002a	0.006 ± 0.001a	0.021 ± 0.008ab	0.025 ± 0.009b	0.045
SUM SFA	31.542 ± 0.726a	36.413 ± 0.648b*	59.230 ± 2.909b	63.132 ± 9.321b	0.001
SUM MUFA	66.686 ± 0.713b	61.644 ± 0.733a*	30.357 ± 1.006a	25.456 ± 6.819a	0.001
SUM PUFA	1.390 ± 0.109a	1.276 ± 0.198a	8.318 ± 1.165b	8.218 ± 2.215b	0.006
SUM n-6 PUFA	0.485 ± 0.050a	0.501 ± 0.091a	2.486 ± 0.325b	2.424 ± 0.670b	0.008
SUM n-3 PUFA	0.350 ± 0.056a	0.322 ± 0.081a	3.157 ± 0.489b	2.563 ± 0.637b	0.007
UFA/SFA	2.168 ± 0.073b	1.732 ± 0.047a*	0.659 ± 0.066a	0.591 ± 0.196a	0.001
n-3/n-6 PUFA	0.706 ± 0.052ab	0.614 ± 0.048a	1.313 ± 0.258c	1.111 ± 0.131bc	0.010
SUM DMA	0.383 ± 0.031a	0.667 ± 0.249ab	2.095 ± 0.915bc	3.194 ± 0.926c	0.014

0 % 100

FA	Ctrl cells (Mol-%)	EPA cells (Mol-%)	Ctrl EVs (Mol-%)	EPA EVs (Mol-%)	P
12:0	0.209 ± 0.030a	0.233 ± 0.020a	1.110 ± 0.131b	1.722 ± 0.865b	0.008
14:0	2.879 ± 0.273a	3.021 ± 0.201a	6.044 ± 0.682b	3.783 ± 0.363ab*	0.020
14:1n-9	0.018 ± 0.005a	0.014 ± 0.008a	0.460 ± 0.23b	0.286 ± 0.163b	0.006
14:1n-7	0.035 ± 0.004a	0.028 ± 0.005a	0.623 ± 0.344b	0.767 ± 0.42b	0.008
14:1n-5	1.213 ± 0.142	1.060 ± 0.269	0.640 ± 0.157	0.561 ± 0.202	0.119
15:0 iso	0.017 ± 0.003a	0.283 ± 0.260ab	0.537 ± 0.330b	0.949 ± 0.495b	0.024
15:0	0.095 ± 0.018a	0.104 ± 0.017a	0.416 ± 0.091b	0.624 ± 0.208b	0.008
15:1n-6	0.012 ± 0.002a	0.029 ± 0.011ab	0.830 ± 0.327bc	4.504 ± 2.834c	0.005
16:0 iso	0.019 ± 0.003a	0.019 ± 0.003a	0.726 ± 0.354b	0.743 ± 0.325b	0.008
DMA 16:0	0.256 ± 0.031	0.291 ± 0.038	0.753 ± 0.247	0.478 ± 0.124	0.161
16:0	27.881 ± 0.459	28.074 ± 0.351	31.165 ± 4.660	30.959 ± 2.003	0.535
16:1n-9	0.577 ± 0.105	0.391 ± 0.052	0.379 ± 0.186	0.237 ± 0.060	0.142
16:1n-7	31.292 ± 1.699bc	32.465 ± 0.732c	2.395 ± 0.891ab	1.098 ± 0.203a	0.007
16:1n-5	0.055 ± 0.017a	0.057 ± 0.010a	0.951 ± 0.231b	0.535 ± 0.200b	0.007
17:0 iso	0.042 ± 0.006a	0.057 ± 0.015a	0.398 ± 0.186ab	0.714 ± 0.135b	0.040
17:0 anteiso	0.031 ± 0.006a	0.030 ± 0.006a	0.523 ± 0.128b	0.443 ± 0.076b	0.008
17:0	0.063 ± 0.012a	0.068 ± 0.012a	1.348 ± 0.388b	2.977 ± 2.154b	0.009
17:1n-8	0.192 ± 0.034	0.198 ± 0.030	0.521 ± 0.256	0.812 ± 0.364	0.406
18:0 iso	0.038 ± 0.012a	0.031 ± 0.006a	1.255 ± 0.464b	0.726 ± 0.202b	0.008
DMA 18:0	0.093 ± 0.011	0.115 ± 0.016	0.339 ± 0.121	0.415 ± 0.186	0.071
DMA 18:1n-9	0.063 ± 0.007a	0.058 ± 0.009a	1.041 ± 0.584b	0.584 ± 0.244ab	0.047
DMA 18:1n-7	0.096 ± 0.014a	0.114 ± 0.015a	0.820 ± 0.290b	0.792 ± 0.195b	0.007
18:0	1.846 ± 0.249a	1.784 ± 0.189ab	12.902 ± 2.655bc	19.977 ± 2.704c	0.007
18:1n-9	21.050 ± 2.665	19.511 ± 1.735	13.915 ± 5.069	12.054 ± 6.321	0.552
18:1n-7	8.966 ± 0.568c	8.383 ± 0.591bc	2.517 ± 0.529ab	1.802 ± 0.589a	0.008
18:1n-5	0.590 ± 0.066	0.521 ± 0.039	0.355 ± 0.213	0.357 ± 0.096	0.358
18:2n-6	0.120 ± 0.023a	0.115 ± 0.018a	0.919 ± 0.089b	0.792 ± 0.316b	0.034
18:3n-6	0.007 ± 0.002a	0.011 ± 0.003a	0.302 ± 0.163b	0.299 ± 0.105b	0.007
19:0	0.012 ± 0.005ab	0.01 ± 0.005a	0.875 ± 0.593c	0.149 ± 0.053bc	0.010
19:1n-10	0.026 ± 0.009a	0.026 ± 0.005a	1.226 ± 0.708b	0.480 ± 0.115b	0.007
19:1n-8	0.040 ± 0.013a	0.041 ± 0.014a	1.152 ± 0.844b	0.344 ± 0.097b	0.016
18:3n-3	0.012 ± 0.004a	0.015 ± 0.003a	0.727 ± 0.283b	0.877 ± 0.415b	0.008
20:0	0.051 ± 0.006ab	0.032 ± 0.005a*	0.279 ± 0.030b	0.281 ± 0.044b	0.004
20:1n-9	0.198 ± 0.029a	0.189 ± 0.024a	0.634 ± 0.133b	0.348 ± 0.102ab	0.030
20:1n-7	0.165 ± 0.026	0.162 ± 0.023	0.643 ± 0.269	0.300 ± 0.121	0.637
20:2n-9	0.072 ± 0.006a	0.065 ± 0.011a	0.700 ± 0.299b	0.382 ± 0.154ab	0.046
20:2n-6	0.008 ± 0.001a	0.009 ± 0.003a	0.109 ± 0.055b	0.099 ± 0.021b	0.016
20:3n-9	0.339 ± 0.022	0.245 ± 0.067	0.476 ± 0.281	0.222 ± 0.082	0.569
20:3n-6	0.025 ± 0.006a	0.030 ± 0.005ab	0.789 ± 0.287c	0.466 ± 0.229bc	0.011
20:4n-6	0.273 ± 0.062	0.264 ± 0.071	0.396 ± 0.148	0.511 ± 0.199	0.651
20:3n-3	0.007 ± 0.002a	0.006 ± 0.001a	0.072 ± 0.028b	0.095 ± 0.059b	0.011
20:4n-3	0.011 ± 0.004a	0.010 ± 0.003a	0.105 ± 0.065b	0.152 ± 0.079b	0.008
20:5n-3	0.054 ± 0.011	0.433 ± 0.122	0.318 ± 0.133	0.787 ± 0.650	0.088
22:0	0.052 ± 0.011ab	0.036 ± 0.010a	0.165 ± 0.047bc	0.328 ± 0.106c	0.008
22:1n-9	0.031 ± 0.008ab	0.019 ± 0.005a	0.181 ± 0.071b	0.145 ± 0.068ab	0.042
22:1n-7	0.011 ± 0.002ab	0.006 ± 0.001a	0.073 ± 0.031b	0.066 ± 0.029b	0.012
22:2n-9	0.006 ± 0.002a	0.010 ± 0.002ab	0.053 ± 0.017b	0.047 ± 0.014b	0.007
22:3n-9	0.116 ± 0.024ab	0.042 ± 0.024a	1.755 ± 0.651c	1.227 ± 0.421bc	0.005
23:0	0.116 ± 0.041	0.152 ± 0.051	0.755 ± 0.693	0.158 ± 0.096	0.897
22:4n-6	0.123 ± 0.051	0.127 ± 0.036	0.261 ± 0.092	0.281 ± 0.167	0.640
22:5n-6	0.025 ± 0.006	0.022 ± 0.006	0.135 ± 0.056	0.231 ± 0.190	0.226
22:4n-3	0.006 ± 0.001a	0.006 ± 0.001a	0.059 ± 0.013b	0.079 ± 0.053ab	0.040
22:5n-3	0.165 ± 0.047a	0.687 ± 0.190b*	0.779 ± 0.426b	0.598 ± 0.150b	0.043
24:0	0.046 ± 0.011	0.051 ± 0.009	0.182 ± 0.060	0.453 ± 0.286	0.089
22:6n-3	0.193 ± 0.063a	0.195 ± 0.056a	2.785 ± 1.223b	0.589 ± 0.23ab	0.040
24:1n-9	0.051 ± 0.013	0.041 ± 0.009	0.085 ± 0.026	0.236 ± 0.137	0.220
24:1n-7	0.009 ± 0.004a	0.007 ± 0.001a	0.046 ± 0.020b	0.077 ± 0.050b	0.021
SUM SFA	33.398 ± 0.507a	33.984 ± 0.527ab	58.680 ± 3.794cb	64.987 ± 4.693c	0.007
SUM MUFA	64.531 ± 0.484c	63.147 ± 0.547bc	27.627 ± 2.818ab	25.009 ± 4.044a	0.006
SUM PUFA	1.564 ± 0.295a	2.290 ± 0.315ab	10.739 ± 1.919c	7.735 ± 2.625bc	0.005
SUM n-6 PUFA	0.582 ± 0.142a	0.577 ± 0.128a	2.911 ± 0.663b	2.679 ± 0.645b	0.009
SUM n-3 PUFA	0.449 ± 0.128a	1.351 ± 0.190ab*	4.844 ± 1.913b	3.178 ± 1.541b	0.013
UFA/SFA	1.982 ± 0.044c	1.928 ± 0.043bc	0.667 ± 0.103ab	0.527 ± 0.105a	0.007
n-3/n-6 PUFA	0.739 ± 0.045a	2.487 ± 0.228b*	1.498 ± 0.417ab	1.022 ± 0.231a	0.016
SUM DMA	0.507 ± 0.058a	0.579 ± 0.071a	2.954 ± 1.045b	2.269 ± 0.547b	0.007

0 % 100

Figure S5. Fatty acid (FA) profiles of Simpson Golabi Behmel Syndrome (SGBS) cells and extracellular vesicles (EVs) from treatments. FAs of mature cells and EVs from tumor necrosis factor α (TNFα), palmitic acid (PA, 16:0), and eicosapentaenoic acid (EPA, 20:5n-3) treatments were determined from total lipids with gas chromatography–mass spectrometry. Results are presented as mol-%, mean ± SEM. FAs are listed in the order of increasing chromatographic retention time. DMA = plasmalogen alkenyl chain-derived dimethyl acetal derivative, SFA = saturated fatty acid, MUFA = monounsaturated fatty acid, PUFA = polyunsaturated fatty acid, unsaturated FA (UFA) = MUFA + PUFA. Means with no common letter are significantly different from each other within a row (Kruskal–Wallis ANOVA). * $p \leq 0.05$ Mann–Whitney U test *vs*. control. The results of TNFα and PA treatments were measured from 5 independent experiments and the results of EPA treatments from 7 independent experiments.

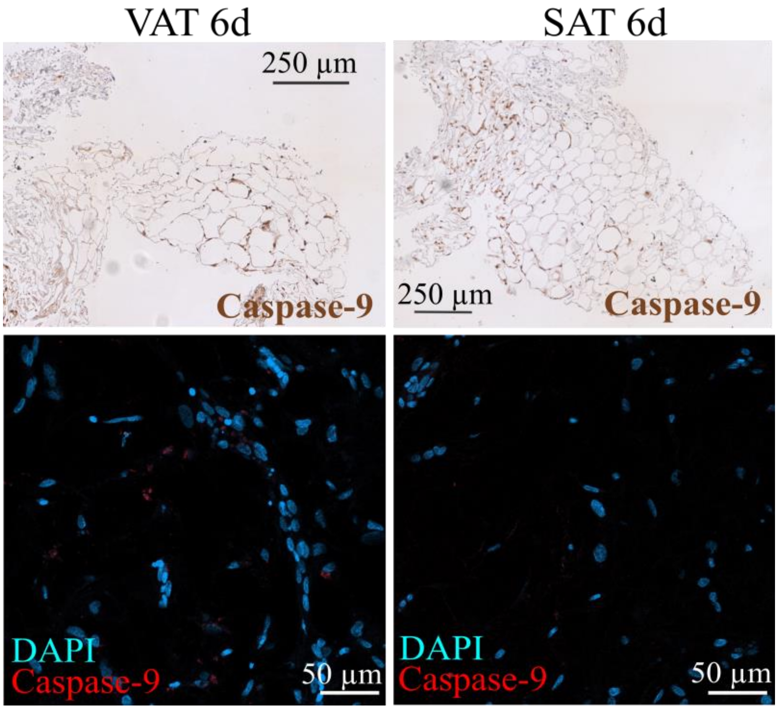

Figure S6. Caspase-9 staining of patient adipose tissue (AT) samples. AT samples from bariatric surgeries were cultured *ex vivo* in extracellular vesicle (EV)-free culture medium for several days, after which EVs were isolated by differential steps of ultracentrifugation. After 6 days of culture initiation, paraffin sections of both visceral (VAT) and subcutaneous (SAT) ATs were obtained and stained both immunohistochemically and fluorescently for caspase-9, in order to confirm that no major degree of apoptosis is present in AT cultures.

FA	VAT *ex vivo* medium (Mol-%)	SAT *ex vivo* medium (Mol-%)	VAT EVs (Mol-%)	SAT EVs (Mol-%)	*P*
12:0	1.311 ± 0.394	2.720 ± 0.762	1.012 ± 0.465	2.078 ± 1.110	0.415
14:0	4.303 ± 0.321	3.442 ± 0.317	3.805 ± 0.684	2.601 ± 0.359	0.078
14:1n-9	0.269 ± 0.062	0.185 ± 0.045	0.255 ± 0.122	0.216 ± 0.057	0.849
14:1n-7	0.190 ± 0.043	0.194 ± 0.046	0.455 ± 0.334	0.776 ± 0.384	0.711
14:1n-5	0.535 ± 0.059	0.697 ± 0.121	0.595 ± 0.268	0.765 ± 0.120	0.442
15:0 iso	0.176 ± 0.030	0.305 ± 0.087	0.460 ± 0.359	0.981 ± 0.503	0.258
15:0	0.343 ± 0.053	0.354 ± 0.024	0.333 ± 0.141	0.254 ± 0.082	0.527
15:1n-6	0.326 ± 0.059	0.319 ± 0.064	0.751 ± 0.446	1.358 ± 0.401	0.072
16:0 iso	0.180 ± 0.025	0.244 ± 0.045	0.234 ± 0.109	0.334 ± 0.095	0.447
DMA 16:0	0.711 ± 0.075	0.687 ± 0.130	0.688 ± 0.138	1.108 ± 0.198	0.298
16:0	28.100 ± 1.202	24.230 ± 2.185	25.333 ± 4.289	24.200 ± 2.105	0.357
16:1n-9	0.333 ± 0.057	0.492 ± 0.085	0.269 ± 0.044	0.204 ± 0.086	0.154
16:1n-7	1.860 ± 0.116	2.095 ± 0.337	1.481 ± 0.409	1.525 ± 0.570	0.687
16:1n-5	0.434 ± 0.058	0.297 ± 0.047	0.431 ± 0.238	0.621 ± 0.219	0.321
17:0 iso	1.110 ± 0.132	0.721 ± 0.154	0.811 ± 0.248	1.063 ± 0.273	0.209
17:0 anteiso	0.399 ± 0.036	0.452 ± 0.031	0.282 ± 0.012	0.562 ± 0.209	0.137
17:0	0.624 ± 0.046	1.440 ± 0.455*	1.197 ± 0.883	1.072 ± 0.589	0.154
17:1n-8	0.236 ± 0.028	0.269 ± 0.043	0.284 ± 0.132	0.242 ± 0.044	0.964
18:0 iso	0.943 ± 0.145	0.924 ± 0.225	1.098 ± 0.476	1.890 ± 0.399	0.289
DMA 18:0	0.341 ± 0.050	0.309 ± 0.080	0.438 ± 0.166	0.412 ± 0.189	0.737
DMA 18:1n-9	0.888 ± 0.215	0.676 ± 0.198	1.158 ± 0.853	1.898 ± 0.903	0.391
DMA 18:1n-7	1.241 ± 0.205	0.913 ± 0.339	1.126 ± 0.528	1.598 ± 0.687	0.675
18:0	15.560 ± 1.189	14.047 ± 1.744	15.636 ± 1.519	17.878 ± 1.911	0.593
18:1n-9	23.872 ± 0.652b	21.593 ± 1.087ab	18.659 ± 4.619ab	13.976 ± 2.147a	0.037
18:1n-7	3.179 ± 0.123b	3.080 ± 0.247b	2.363 ± 0.301ab	1.018 ± 0.435a*	0.022
18:1n-5	0.429 ± 0.045	0.360 ± 0.050	0.326 ± 0.070	0.202 ± 0.027	0.088
18:2n-6	2.445 ± 0.330b	2.875 ± 0.539b	1.310 ± 0.479ab	1.002 ± 0.212a	0.033
18:3n-6	0.265 ± 0.065	0.913 ± 0.469	0.301 ± 0.134	0.970 ± 0.419	0.509
19:0	1.235 ± 0.395	1.353 ± 0.491	1.344 ± 0.767	0.600 ± 0.114	0.795
19:1n-10	0.479 ± 0.030	0.695 ± 0.179	1.210 ± 0.946	1.030 ± 0.147	0.169
19:1n-8	0.176 ± 0.018	0.657 ± 0.312	0.344 ± 0.151	0.841 ± 0.160*	0.060
18:3n-3	0.220 ± 0.028	0.400 ± 0.093	0.730 ± 0.291	0.697 ± 0.220	0.079
20:0	0.226 ± 0.015	0.168 ± 0.028	0.139 ± 0.040	0.174 ± 0.071	0.267
20:1n-9	0.270 ± 0.032	0.884 ± 0.482	1.862 ± 1.642	0.554 ± 0.364	0.994
20:1n-7	0.119 ± 0.021	0.312 ± 0.186	0.735 ± 0.587	0.228 ± 0.035	0.210
20:2n-9	0.146 ± 0.070	0.401 ± 0.183	0.456 ± 0.326	0.153 ± 0.031	0.507
20:2n-6	0.173 ± 0.028	0.221 ± 0.083	0.266 ± 0.165	0.352 ± 0.104	0.571
20:3n-9	0.114 ± 0.015	0.642 ± 0.481*	1.556 ± 1.018	0.501 ± 0.380	0.256
20:3n-6	0.507 ± 0.107	0.796 ± 0.178	0.909 ± 0.368	1.109 ± 0.300	0.382
20:4n-6	0.856 ± 0.246	1.539 ± 0.366	0.520 ± 0.110	0.602 ± 0.224	0.200
20:3n-3	0.044 ± 0.010	0.068 ± 0.025	0.058 ± 0.021	0.046 ± 0.010	0.910
20:4n-3	0.056 ± 0.010	0.069 ± 0.025	0.082 ± 0.050	0.058 ± 0.030	0.993
20:5n-3	0.386 ± 0.084	0.427 ± 0.076	0.501 ± 0.216	0.920 ± 0.332	0.361
22:0	0.263 ± 0.031	0.157 ± 0.029*	0.115 ± 0.017	0.160 ± 0.117	0.097
22:1n-9	0.103 ± 0.026	0.233 ± 0.089	0.029 ± 0.011	0.150 ± 0.041*	0.100
22:1n-7	0.048 ± 0.006	0.056 ± 0.025	0.017 ± 0.003	0.081 ± 0.029*	0.055
22:2n-9	0.044 ± 0.010	0.049 ± 0.018	0.041 ± 0.030	0.076 ± 0.024	0.609
22:3n-9	1.130 ± 0.218	1.278 ± 0.432	0.824 ± 0.265	3.926 ± 2.521	0.629
23:0	0.060 ± 0.008	0.378 ± 0.231	0.038 ± 0.011	0.145 ± 0.046*	0.143
22:4n-6	0.093 ± 0.025	0.487 ± 0.388	0.114 ± 0.076	0.263 ± 0.120	0.390
22:5n-6	0.160 ± 0.024	0.132 ± 0.024	0.380 ± 0.167	0.344 ± 0.073	0.147
22:4n-3	0.030 ± 0.005	0.032 ± 0.016	0.039 ± 0.018	0.076 ± 0.035	0.158
22:5n-3	1.000 ± 0.217	1.272 ± 0.306	0.905 ± 0.347	1.610 ± 0.719	0.810
24:0	0.116 ± 0.015	0.130 ± 0.030	0.131 ± 0.059	0.166 ± 0.017	0.656
22:6n-3	1.149 ± 0.304	2.166 ± 0.470	5.424 ± 3.951	4.081 ± 1.244	0.145
24:1n-9	0.152 ± 0.033	0.137 ± 0.022	0.099 ± 0.039	0.198 ± 0.024	0.261
24:1n-7	0.046 ± 0.016	0.030 ± 0.006	0.045 ± 0.023	0.054 ± 0.018	0.762
SUM SFA	54.948 ± 1.528	51.064 ± 3.140	51.965 ± 3.876	54.159 ± 2.104	0.733
SUM MUFA	33.055 ± 0.417	32.583 ± 1.444	30.207 ± 4.497	24.040 ± 3.067	0.102
SUM PUFA	8.816 ± 1.292	13.769 ± 2.361	14.418 ± 5.457	16.786 ± 2.990	0.234
SUM n-6 PUFA	4.498 ± 0.694	6.964 ± 1.414	3.800 ± 0.479	4.642 ± 0.849	0.642
SUM n-3 PUFA	2.884 ± 0.438	4.435 ± 0.864	7.740 ± 4.535	7.488 ± 1.358	0.129
UFA/SFA	0.772 ± 0.054	0.949 ± 0.112	0.875 ± 0.113	0.757 ± 0.036	0.649
n-3/n-6 PUFA	0.658 ± 0.063	0.660 ± 0.082	1.815 ± 0.909	1.670 ± 0.357	0.054
SUM DMA	3.181 ± 0.307	2.584 ± 0.528	3.410 ± 1.601	5.015 ± 1.797	0.423

0 % 100

Figure S7. Fatty acid (FA) profiles of adipose tissue (AT) extracellular vesicles (EVs) and culture media. Visceral (VAT) and subcutaneous adipose tissue (SAT) EVs, as well as *ex vivo* culture media were determined from total lipids with gas chromatography–mass spectrometry. Results are presented as mol-%, mean ± SEM. FAs are listed in the order of increasing chromatographic retention time. DMA = plasmalogen alkenyl chain-derived dimethyl acetal derivative, SFA = saturated fatty acid, MUFA = monounsaturated fatty acid, PUFA = polyunsaturated fatty acid, unsaturated fatty acid (UFA) = MUFA + PUFA. Means with no common letter are significantly different from each other within a row (Kruskal–Wallis ANOVA). * $p \leq 0.05$ Mann–Whitney U test *vs*. VAT. Results of FA profiles were analyzed from 3 EV samples, from which each corresponded to a pooled EV sample from 2 different patients. Media samples were obtained from 2 patient's AT *ex vivo* cultures.

Supplementary methods

Immunofluorescent and immunohistochemical staining of adipose tissue (AT) for caspase-9

AT samples were fixed in 10% buffered formalin solution and embedded in paraffin. For caspase-9 immunohistochemical staining, 5 µm thick sections were deparaffinized, and antigen retrieval in 10 mM citrate buffer (pH 6.0) in a pressure cooker for 10 min was performed. Endogenous peroxidase activity was blocked with 1% H_2O_2 for 5 min, and samples were washed with H_2O 2 × 3 min and PBS 2 × 5 min. For blocking non-specific antibody binding, sections were incubated with 1% bovine serum albumin (BSA) PBS for 30 min. For primary antibody binding, sections were incubated with anti-pro caspase-9 phospho-S196 antibody (ab135544, Abcam, Cambridge, UK) 1:100 1% BSA PBS dilution at +4°C for o/n. Samples were washed with PBS 3 × 10 min and incubated with the secondary antibody, biotinylated anti-rabbit IgG (BA-1000, Vector Laboratories, Newark, CA, USA) 1:150 in 1% BSA PBS at RT for 1 h. Caspase-9 antibody was visualized with Vectastain Elite® ABC kit (PK-6101, Vector Laboratories) using 3,3'-diaminobenzidine to stain in brown, and Mayer's hematoxylin to the detection of nuclei in blue. Samples were imaged with Leica Thunder imager 3D Tissue Slide scanner with 10× dry objective (Leica Microsystems, Wetzlar, Germany).

For immunofluorescent staining, the sections were deparaffinized, and autofluorescence was removed by 50 mM glycine in H_2O for 40 min. For blocking non-specific antibody binding, sections were incubated with 1% BSA PBS for 30 min at +37°C. Primary antibody staining was performed as above. After 3 × 5 min phosphate buffer (PB) washes, secondary antibody staining with Texas Red anti-rabbit IgG (TI-1000, Vector Laboratories) dilution 1:200 in PB at RT for 1 h was performed. After 3 × 5 min PB washes, nuclei were stained with 1 µg/ml 4´,6-diamidino-2-phenylindole (DAPI, D8417, Sigma-Aldrich, St. Louis, MO, USA). Sections were then mounted in Vectashield (H-1000, Vector Laboratories), and imaged with 40× NA 1.3 objective on a Zeiss Axio Observer inverted microscope equipped with a Zeiss LSM 800 confocal module (Carl Zeiss Microimaging GmbH, Jena, Germany).

Propidium iodide (PI) staining of Simpson Golabi Behmel Syndrome (SGBS) cells for confocal microscopy

SGBS cells were plated and differentiated on Ibidi chamber slides (Ibidi GmbH, Martinsried, Germany), and then treated with TNFα or PA for 24h. To demonstrate cell viability, live SGBS cells were first stained with NucBlue® Live Cell Stain ReadyProbesTM reagent (R37605, Life Technologies, Eugene, OR, USA) at RT for 5 min. Cells were then stained with 60 µM PI (SC-3541, Santa Cruz Biotechnology, Santa Cruz, CA, USA) at +37°C for 5 min and washed with PBS. Cells were visualized with 40× NA 1.3 objective on a Zeiss Axio Observer inverted microscope equipped with a Zeiss LSM 800 confocal module. PI-positive cells were manually counted from confocal images, and the data were expressed as % PI-positive cells from counted cells.
